# Coordination‐Asymmetry‐Regulated Reconstruction of Electron‐Rich Cu Sites for pH‐Dependent Nitrate‐to‐Ammonia Electrocatalysis

**DOI:** 10.1002/advs.78164

**Published:** 2026-10-14

**Authors:** Shih‐Ching Huang, Yu‐Ru Lin, Hsiang‐Chun Yu, Yi‐Dong Lin, Yu‐Chiao Chang, Chun‐Kuo Peng, Yu‐Chang Lin, Wen‐Wei Wu, Hao Ming Chen, Chi‐Liang Chen, Han‐Yi Chen, Yan‐Gu Lin

**Affiliations:** ^1^ Scientific Research Division National Synchrotron Radiation Research Center Hsinchu Taiwan; ^2^ Department of Chemistry National Taiwan University Taipei Taiwan; ^3^ Department of Materials Science and Engineering National Yang Ming Chiao Tung University Hsinchu Taiwan; ^4^ Department of Materials Science and Engineering National Tsing Hua University Hsinchu Taiwan; ^5^ Experimental Facility Division National Synchrotron Radiation Research Center Hsinchu Taiwan

**Keywords:** active‐site reconstructions, coordination asymmetry, in situ/operando, nitrate‐to‐ammonia electrocatalysis, pH‐universal, reaction intermediates, single‐atom catalysts

## Abstract

Electrocatalytic nitrate reduction reaction (*e‐*NO_3_RR) offers a promising route to valorize nitrate‐contaminated wastewater while enabling sustainable ammonia production; however, how local coordination asymmetry regulates Cu‐site evolution and reaction pathways across different pH environments remains insufficiently understood. Herein, three‐ and four‐nitrogen‐coordinated Cu single‐atom motifs, denoted as Cu─N_3_ and Cu─N_4_, are constructed as well‐defined pre‐catalyst models to elucidate how the initial Cu coordination environment directs pH‐dependent active‐site reconstruction. *Operando* X‐ray absorption spectroscopy and wavelet‐transform EXAFS reveal that Cu─N_4_ readily undergoes rapid reconstruction under acidic, neutral, and alkaline conditions, generating low‐coordinated, electron‐enriched Cu species while concomitantly inducing Cu─Cu aggregation and excessive Cu─site reduction. In contrast, Cu─N_3_ displays more regulated site evolution, with limited activation under acidic conditions but progressive formation of low‐coordinated Cu─N_x_ active structures under neutral and alkaline conditions. In situ ATR‐FTIR further verifies that the dynamically evolved Cu─N_3_ sites enhance nitrate adsorption, facilitate N─O bond activation, and promote the formation of hydrogenated nitrogen intermediates. These findings establish coordination‐governed site evolution as a design principle for balancing productive Cu─site activation and metal‐centered over‐reduction, providing molecular‐level guidance for the design of pH‐adaptive NO_3_RR catalysts.

## Graphical abstract

Distinct initial Cu coordination environments drive different pH‐dependent structural evolution pathways, resulting in divergent nitrate reduction activity and behavior.

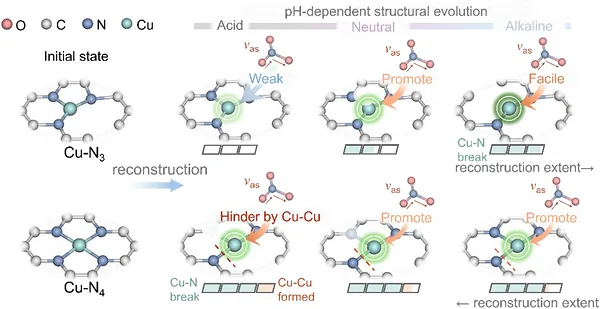

## 1 Introduction

Ammonia is an indispensable chemical for modern agriculture and industry and has also been regarded as a promising carbon‐free energy carrier [1, 2, 3]. Compared with the energy‐intensive Haber‐Bosch process, which relies on high temperatures and high pressures, the electrocatalytic nitrate reduction reaction (*e‐*NO_3_RR) provides an attractive route to produce ammonia under mild conditions while simultaneously enabling the remediation and valorization of nitrate‐contaminated wastewater. However, *e‐*NO_3_RR is a complex multielectron and multiproton process with intricate reaction mechanisms [4]. Its activity and selectivity are therefore highly sensitive to the electrolyte environment. Recent studies have further highlighted the coupled importance of nitrogen‐oxyanion activation and active‐hydrogen‐mediated hydrogenation in governing the overall reduction process [5, 6, 7]. In particular, pH can regulate nitrate activation, proton/electron transfer, active hydrogen *H‐mediated hydrogenation [3, 8], competing hydrogen evolution, and the structural evolution of catalytic sites. Because practical nitrate‐containing wastewater may involve broad and fluctuating pH conditions, understanding how pH governs both the reaction pathway and the dynamic evolution of active sites is essential for the rational design of practical nitrate electroreduction catalysts [9].

Recent advances in Cu‐based electrocatalysts have demonstrated the importance of regulating the local catalytic environment for nitrate reduction and the subsequent conversion of nitrogenous intermediates [10, 11, 12]. Cu single‐atom catalysts have attracted increasing attention for *e‐*NO_3_RR because their atomically dispersed Cu centers provide tunable coordination environments and well‐defined local electronic structures [13, 14]. Compared with conventional Cu‐based catalysts, Cu single‐atom sites offer a more precise platform for correlating metal coordination, electronic structure, intermediate adsorption, and catalytic behavior. Previous studies have modulated Cu single‐atom sites through different coordination motifs, including Cu─N_4_, low‐coordinated Cu─N_x_, and heteroatom‐modified Cu coordination environments [13, 15, 16, 17, 18, 19, 20, 21, 22], demonstrating that local coordination strongly influences nitrate adsorption [23, 24, 25], intermediate binding, and ammonia selectivity. These advances highlight coordination engineering as an effective strategy for tuning nitrate reduction performance. Beyond ligand identity alone, these coordination modifications can alter the coordination number, geometric arrangement, and local symmetry of the metal center, with coordination symmetry representing an important structural dimension governing its geometric and electronic characteristics [26]. In particular, symmetry lowering can arise when changes in ligand identity, coordination number, or local geometry create inequivalent coordination environments around the single‐atom center [27, 28]. Recent studies have extended asymmetric coordination engineering to diverse Cu single‐atom motifs, where variations in the local ligand environment and coordination geometry have been exploited to tune the electronic properties of Cu centers and their interactions with nitrate‐derived species [13, 15, 22, 29]. Nevertheless, most reported structure‐activity correlations are still established based on the as‐prepared catalyst structure [15, 16, 30, 31]. Under working *e‐*NO_3_RR conditions, however, the genuine Cu active structure may differ substantially from its initial coordination state. *Operando* studies have shown that Cu─N_4_ single‐atom sites can undergo potential‐driven restructuring during nitrate electroreduction, leading to the formation of Cu clusters or nanoparticles [14]. Similar dynamic coordination evolution has also been observed for other transition‐metal single‐atom sites, such as Fe‐N_4_ to Fe‐N_3_ transformation under *e‐*NO_3_RR conditions [32]. These findings suggest that metal single‐atom sites should be considered dynamic pre‐catalysts rather than static active structures.

Despite these advances, how the initial Cu coordination motif governs pH‐dependent active‐site reconstruction, productive active‐site formation, and metal‐centered over‐reduction during *e‐*NO_3_RR remains insufficiently understood. This issue is particularly important because reductive coordination reconstruction can be either beneficial or detrimental, depending on its rate and extent. Moderate reconstruction may generate low‐coordinated, electron‐enriched Cu active sites that facilitate nitrate adsorption, N─O bond activation, and subsequent hydrogenation. In contrast, excessively rapid or excessive Cu‐site reduction may induce Cu─Cu aggregation and metallic Cu formation [14, 33, 34, 35], thereby altering the nature of the catalytic interface and compromising the structural definition of the active sites. Therefore, a central challenge is to establish a direct relationship among the initial coordination motif, pH‐dependent coordination evolution, and the genuine Cu active structures formed under operating *e‐*NO_3_RR conditions. Addressing this challenge requires in situ characterization capable of tracking Cu coordination, electronic structure, and surface intermediates during reaction, rather than relying solely on ex situ structural analysis before and after electrolysis.

In this study, we investigated how pH regulates the in situ evolution pathways of Cu single‐atom sites with different initial coordination environments and how these distinct evolution pathways ultimately govern catalytic performance during *e‐*NO_3_RR. Three‐ and four‐nitrogen‐coordinated Cu single‐atom structures, denoted as Cu─N_3_ and Cu─N_4_, are constructed as well‐defined pre‐catalyst models to investigate how the initial Cu coordination environment controls active‐site evolution during *e‐*NO_3_RR. By regulating the coordination number within the same N‐ligand framework, these two motifs provide a simplified model system with distinct initial coordination structures and local symmetries, while limiting variations associated with different ligand identities and enabling their influence on subsequent reconstruction pathways to be examined more directly. By combining electrochemical measurements, in situ Cu K‐edge X‐ray absorption spectroscopy (XAS), wavelet‐transform EXAFS (WT‐EXAFS), and in situ attenuated total reflection Fourier transform infrared (ATR‐FTIR) spectroscopy, we track the coordination, electronic, and interfacial evolution of Cu sites under acidic, neutral, and alkaline *e‐*NO_3_RR conditions. The results reveal that Cu─N_4_ readily undergoes rapid coordination reconstruction across all three pH environments. Although this process can generate low‐coordinated, electron‐enriched Cu active sites, it is accompanied by pronounced Cu─Cu aggregation and excessive Cu‐site reduction. In contrast, Cu─N_3_ exhibits more regulated site evolution. Under acidic conditions, its reconstruction is restricted, resulting in insufficient active‐site activation, whereas under neutral and alkaline conditions, it progressively evolves into low‐coordinated Cu─N_x_ active structures that promote nitrate adsorption, N─O bond activation, and hydrogenated intermediate formation. These findings demonstrate that the initial coordination structure governs the pH‐dependent site evolution of Cu single‐atom pre‐catalysts, thereby determining the balance between productive active‐site formation and metal‐centered over‐reduction. This work provides a dynamic coordination perspective for the rational design of Cu single‐atom catalysts for nitrate‐to‐ammonia electrocatalysis.

## 2 Results and Discussion

### 2.1 Structure Characterization of Cu‐SACs

Cu─N_4_ and Cu─N_3_ single‐atom catalysts (SACs) were structurally characterized to elucidate the coordination‐dependent local structure and electronic environment of the isolated Cu sites. As shown in Figure 1a and Figure S1, high‐angle annular dark‐field scanning transmission electron microscopy (HAADF‐STEM) images reveal numerous isolated bright spots uniformly distributed throughout both Cu─N_4_ and Cu─N_3_, which can be assigned to atomically dispersed Cu species. No obvious Cu aggregates or nanoparticles are observed over a wide field of view, indicating that Cu is highly dispersed in both catalysts. The corresponding intensity profile of representative Cu sites in Cu─N_3_ further shows an angstrom‐scale feature (Figure 1b), supporting the assignment of these bright spots to isolated Cu species.

**FIGURE 1:**
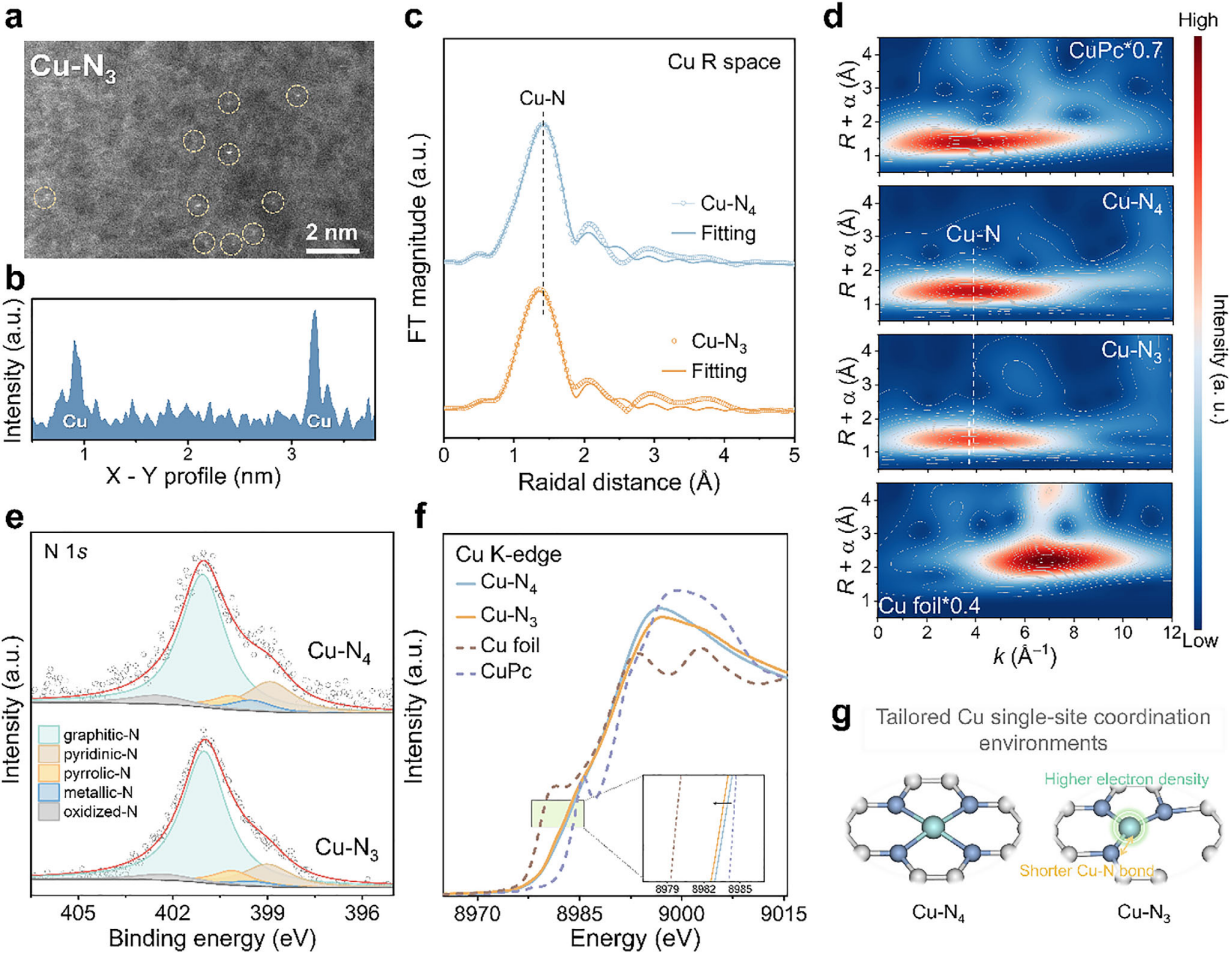
Structure characterization of symmetric and asymmetric Cu single‐atom catalysts. (a) High‐resolution TEM image of the Cu‐N_3_ catalyst and (b) corresponding intensity profile of the isolated Cu site. (c) *k* ^2^‐weighted EXAFS fitting of Cu‐N_4_ and Cu‐N_3_. (d) WT‐EXAFS contour plots at the Cu K‐edges for Cu‐N_4_ and Cu‐N_3_ together with Cu foil and CuPc references. (e) Deconvoluted N 1*s* XPS spectra of Cu‐N_3_ and Cu‐N_4_ catalysts. (f) Cu K‐edge XANES spectra of Cu‐N_4_ and Cu‐N_3_ with Cu foil and CuPc references. (g) Schematic illustration of the tailorable Cu single‐atom sites.

High‐resolution powder X‐ray diffraction (HR‐PXRD) patterns show only broad graphitic carbon features, without detectable reflections corresponding to metallic Cu or copper oxide phases, suggesting the absence of crystalline Cu‐based nanoparticles or CuO_x_ impurities in both samples (Figure S2). In addition, inductively coupled plasma optical emission spectroscopy (ICP‐OES) analysis reveals Cu loading of 0.76 and 0.11 wt.% for Cu─N_4_ and Cu─N_3_ SACs, respectively (Table S1). Together with the HAADF‐STEM observations, these results indicate that the modulation of the local Cu coordination environment does not induce detectable Cu aggregation or crystalline Cu/CuO_x_ formation, supporting the structural integrity of the atomically dispersed Cu sites. The local environment of the isolated Cu sites was further investigated by Cu K‐edge XAS. As shown in the k‐space spectra (Figure S3), both Cu─N_4_ and Cu─N_3_ exhibit relatively simple χ(k) oscillations, primarily arising from first‐shell Cu─N scattering. In contrast to the more complex oscillation features observed for metallic Cu references, no apparent higher‐shell scattering contribution is detected in either catalyst, further supporting the formation of isolated Cu single‐atom sites. The Fourier‐transformed EXAFS spectra and the corresponding fitting results (Figure 1c) show that both Cu─N_4_ and Cu─N_3_ display a dominant first‐shell scattering peak, which is attributed to Cu─N coordination. No significant Cu─Cu scattering feature is observed in the higher‐R region for either sample, excluding the presence of substantial aggregated Cu species within the detection limit of EXAFS. Quantitative EXAFS fitting further reveals distinct local coordination environments for the two catalysts. As summarized in Table S2, Cu─N_4_ SACs exhibit a Cu─N coordination number close to 4, whereas Cu─N_3_ SACs show a reduced Cu─N coordination number of approximately 3. These results confirm that the local Cu coordination environment is successfully regulated from a more saturated Cu─N_4_ configuration to an under‐coordinated Cu─N_3_ motif. Correspondingly, the fitted Cu─N bond lengths are determined to be 1.93 Å for Cu─N_4_ and 1.91 Å for Cu─N_3_, respectively, suggesting that the under‐coordinated Cu─N_3_ motif is accompanied by a slightly shortened Cu─N bond. WT‐EXAFS analysis was further employed to distinguish the local scattering features of the Cu sites. As shown in Figure 1d, both Cu─N_4_ and Cu─N_3_ SACs exhibit only first‐shell Cu─N scattering features, without detectable high‐k Cu─Cu contributions, further supporting the isolated single‐atom configuration of Cu species. Notably, the Cu─N scattering feature of Cu─N_3_ shifts toward a lower k value relative to that of Cu─N_4_, indicating that the reduced coordination number modifies the local geometric and scattering environment around the Cu center. This change is consistent with the formation of a lower‐symmetry Cu─N_3_ structure rather than a fully coordinated Cu─N_4_ configuration. To further clarify the nitrogen coordination environment around Cu, X‐ray photoelectron spectroscopy (XPS) was carried out. Quantitative XPS analysis shows that Cu─N_4_, Cu─N_3_, and Cu─free N‐doped carbon (N─C) possess comparable overall surface compositions dominated by carbon, with N contents of 3.4, 4.9, and 3.4 at.%, respectively (Table S8). Consistently, their C1*s* spectra exhibit highly similar spectral profiles (Figure S19), indicating no pronounced difference in the overall carbon chemical environments among the three samples. The N 1*s* spectra of Cu─N_4_, Cu─N_3_, and N─C can be deconvoluted into pyridinic N, Cu─N, pyrrolic N, graphitic N, and oxidized N components (Figure 1e and Figure S20), and the fitted fractions are summarized in Table S3. All three samples exhibit similar distributions of the major N species, with the graphitic N remaining the predominant component. Compared with Cu─N_4_, Cu─N_3_ shows a decreased Metal‐N‐related component, consistent with the lower Cu─N coordination number obtained from EXAFS fitting. This independently resolved Cu─N component provides complementary chemical evidence for Cu─N bonding and supports different degrees of N coordination around the Cu sites. This result suggests that Cu sites in Cu─N_3_ possess a more unsaturated local coordination environment. Together, these XPS results support the presence of distinct local Cu coordination environments without a pronounced alteration of the overall chemical environment of the N‐doped carbon matrix.

Cu K‐edge X‐ray absorption near‐edge structure (XANES) spectroscopy further revealed the electronic structure of Cu sites. As shown in Figure 1f, the absorption edge of Cu─N_3_ shifts to lower energy relative to that of Cu─N_4_, indicating a lower average oxidation state and higher electron density at the Cu center in Cu─N_3_. Linear fitting of the XANES spectra (Figure S5) estimates the average Cu valence states to be approximately +1.88 for Cu─N_4_ and +1.73 for Cu─N_3_. The lower oxidation state of Cu─N_3_ indicates that reducing the Cu─N coordination number increases the electronic occupancy of the Cu center, giving rise to a more electron‐rich single‐atom site.

Overall, these structural and spectroscopic results confirm the successful construction of atomically dispersed Cu─N_4_ and Cu─N_3_ single‐atom pre‐catalysts with well‐defined local coordination environments. As schematically illustrated in Figure 1g, precise regulation of the Cu coordination number establishes two distinct coordination motifs, corresponding to four‐coordinate Cu─N_4_ and three‐coordinate Cu─N_3_ sites. Compared with Cu─N_4_, the under‐coordinated Cu─N_3_ motif exhibits reduced local coordination symmetry, a slightly shortened Cu─N bond, and a more electron‐rich Cu center. Such coordination‐dependent structural and electronic differences provide an ideal platform for probing how the initial Cu coordination environment governs the dynamic evolution of Cu single‐atom sites during *e‐*NO_3_RR. Notably, because the Cu loadings of Cu─N_4_ and Cu─N_3_ are different, the subsequent catalytic performance is further assessed using ICP‐normalized ammonia production rates and turnover frequency (TOF), providing a consistent comparison of coordination‐dependent activity.

### 2.2 pH‐Dependent Electrocatalytic Nitrate‐to‐Ammonia Performance

The *e‐*NO_3_RR performance of Cu─N_3_ and Cu─N_4_ was systematically investigated over a wide pH range using alkaline (1.0 m KOH), neutral (0.5 m Na_2_SO_4_), and acidic (0.5 m H_2_SO_4_) electrolytes, each containing 0.1 m KNO_3_. The corresponding linear sweep voltammetry (LSV) curves recorded in the absence and presence of nitrate are presented in Figure S6. The N─C control exhibits only a limited nitrate‐related electrochemical response compared with Cu─N_3_ and Cu─N_4_ across all investigated pH conditions, indicating a limited contribution from the N‐doped carbon matrix alone. Under alkaline conditions, both catalysts exhibit a pronounced enhancement in cathodic current upon nitrate addition, suggesting the occurrence of nitrate‐involved cathodic reduction processes. Notably, Cu─N_3_ displays a more positively shifted onset potential than Cu─N_4_, indicating that the low‐coordinated Cu environment facilitates the initiation of nitrate reduction. In addition to the earlier onset potential, Cu─N_3_ maintains a consistently higher nitrate‐induced current response than Cu─N_4_, further suggesting more favorable electrochemical nitrate reduction behavior under alkaline conditions. Upon transitioning from alkaline to neutral and acidic electrolytes, the nitrate‐induced current enhancement progressively diminishes for both catalysts, reflecting the gradual attenuation of the electrocatalytic response toward nitrate reduction in increasingly proton‐rich environments. More importantly, the distinction between Cu─N_3_ and Cu─N_4_ becomes less pronounced as the electrolyte pH decreases. Although Cu─N_3_ still exhibits a slightly higher current response than Cu─N_4_ under neutral conditions, its relative advantage is substantially reduced compared with that observed in alkaline media. Upon further transition to acidic conditions, the electrocatalytic trend is completely reversed, with Cu─N_4_ exhibiting a larger current response than Cu─N_3_.

While the LSV measurements reveal distinct pH‐dependent electrochemical responses toward nitrate reduction, potentiostatic electrolysis was subsequently conducted to quantitatively evaluate NH_3_ production, with the Faradaic efficiency toward NH_3_ (FE_NH3_) and ICP‐normalized NH_3_ yield rate serving as the primary performance metrics. The total charge passed and the corresponding NH_3_ amount obtained at each applied potential under the investigated pH conditions are summarized in Tables S9 and S10. The ICP‐normalized NH_3_ yield rate was calculated by normalizing the amount of produced NH_3_ to the Cu loading determined by ICP‐OES and the electrolysis time, and is expressed as g mg_Cu_ h^−1^. The corresponding chronoamperometric curves of Cu─N_3_ and Cu─N_4_ recorded at various applied potentials across all investigated pH conditions are provided in Figures S7–S9, while the NH_3_ calibration curves established using the indophenol assay are presented in Figure S10. Under alkaline conditions (Figure 2a), Cu─N_3_ exhibits an earlier onset of NH_3_ production than Cu─N_4_ and rapidly achieves an FE_NH3_ of approximately 85%. Consistent with the FE_NH3_ trend, Cu─N_3_ sustains significantly higher ICP‐normalized NH_3_ yield rates throughout the investigated potential range, reaching 37.38 g mg_Cu_ h^−1^ at ‐0.5 V vs. RHE, whereas Cu─N_4_ achieves only 3.25 g mg_Cu_ h^−1^ under the same potential. This result indicates that the superior alkaline *e‐*NO_3_RR performance of Cu─N_3_ is not merely a consequence of total Cu loading, while suggesting a strong dependence of the catalytic activity on the Cu coordination environment.

**FIGURE 2:**
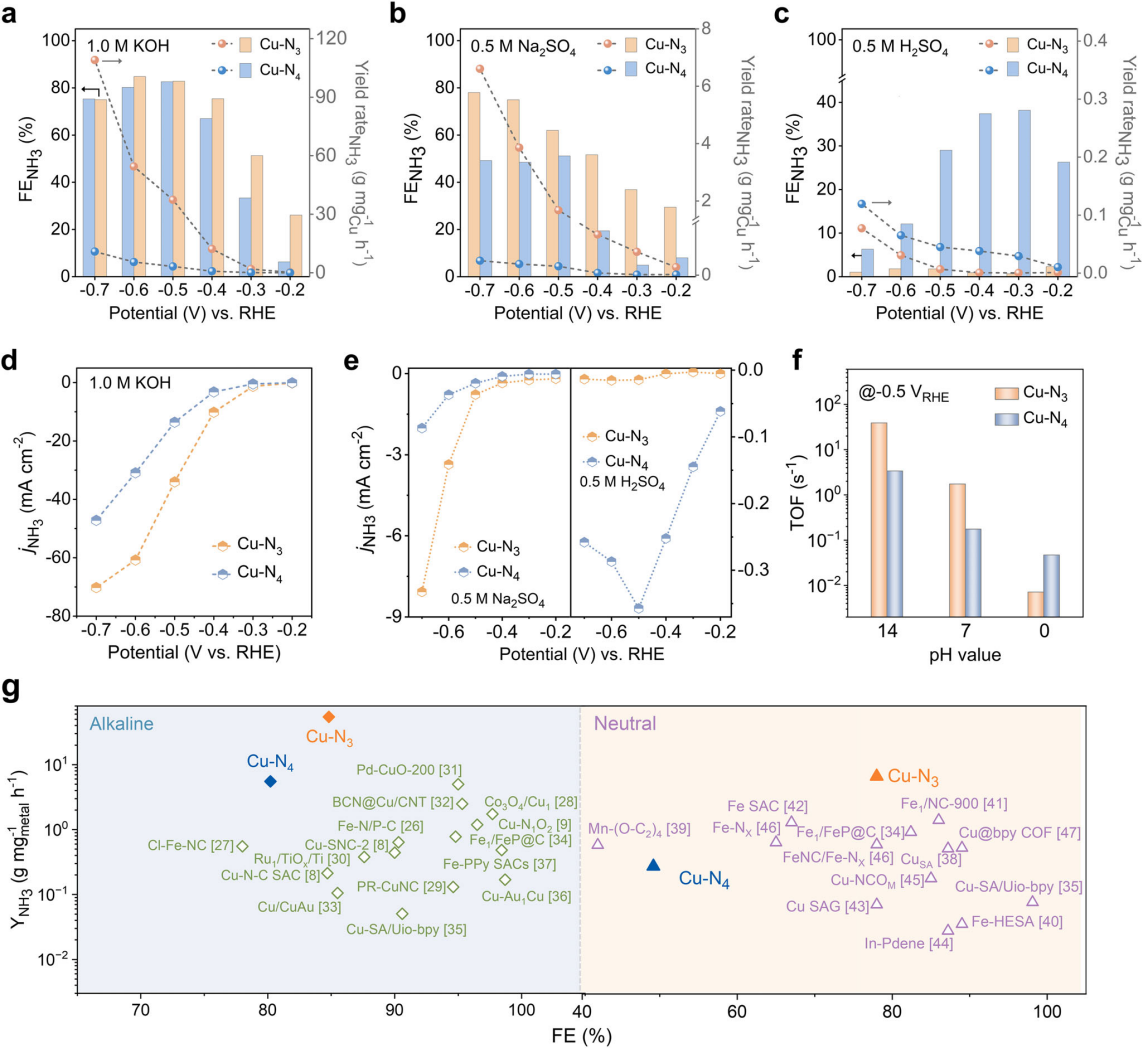
Electrochemical characterization of Cu SAC catalysts under a wide pH range. NH_3_ Faradaic efficiency (FE_NH3_) and yield rate over Cu‐N_3_ and Cu‐N_4_ quantified from ‐0.2 to ‐0.7 V vs. RHE under (a) 1.0 m KOH, (b) 0.5 m Na_2_SO_4_, and (c) 0.5 m H_2_SO_4_ electrolyte containing 0.1 m KNO_3_. NH_3_ partial current density (*j*_NH3_) of Cu‐N_3_ and Cu‐N_4_ under (d) 1.0 m KOH, (e) 0.5 m Na_2_SO_4_ (left panel), and 0.5 m H_2_SO_4_ (right panel) electrolyte containing 0.1 m KNO_3_. (f) Turnover frequency (TOF) of Cu‐N_3_ and Cu‐N_4_ at ‐0.5 V vs. RHE in across various pH conditions. (g) Comparison of nitrate reduction performance of Cu‐N_3_ and Cu‐N_4_ catalysts under alkaline and neutral.

However, this catalytic advantage is not maintained across all electrolyte environments. Reducing the electrolyte pH to neutral conditions (Figure 2b) evidently alters the relative NH_3_ production behavior of Cu─N_3_ and Cu─N_4_. Although Cu─N_3_ continues to exhibit higher FE_NH3_ than Cu─N_4_ across the investigated potential range, the difference in ICP‐normalized NH_3_ yield rate becomes markedly smaller than that observed under alkaline conditions. A further decrease in electrolyte pH to acidic conditions (Figure 2c) results in a reversal of the relative NH_3_ production performance between the two catalysts. Despite the generally suppressed NH_3_ production observed for both catalysts under acidic conditions, Cu─N_4_ exhibits higher FE_NH3_ and ICP‐normalized NH_3_ yield rate than Cu─N_3_ throughout the investigated potential range. To further evaluate the product distribution, the potential‐dependent NO_2_^−^ Faradaic efficiencies (FE_NO2‐_) of both catalysts were quantified using Griess assays (Figures S21 and S22). Under alkaline and neutral conditions, Cu─N_4_ exhibits higher FE_NO2‐_ than Cu─N_3_, while FE_NO2‐_ for both catalysts progressively decreases toward zero at more negative applied potentials. Under acidic conditions, Cu─N_3_ maintains a very low FE_NO2‐_ throughout the investigated potential range, whereas Cu─N_4_ exhibits an FE_NO2‐_ of approximately 10% at less negative potentials, which subsequently decreases toward zero. Notably, the inferior NH_3_ production of Cu─N_3_ under acidic conditions is not accompanied by increased NO_2_^−^ accumulation, indicating that the reversal in NH_3_ performance cannot be simply attributed to a shift in selectivity toward NO_2_^−^ formation. This striking contrast to the alkaline behavior indicates that the relative catalytic advantage of the two coordination environments evolves substantially as the reaction environment becomes increasingly proton‐rich. These observations further suggest that the coordination‐activity relationship cannot be fully captured by FE_NH3_ alone and requires complementary evaluation of productivity and catalytic activity.

To further verify the origin of the detected NH_3_, the NH_4_^+^ background in the KNO_3_‐containing electrolytes was first examined using the indophenol assay. Negligible NH_4_^+^ signals were observed before electrolysis, whereas pronounced signals appeared after electrolysis with Cu─N_3_ and Cu─N_4_ under alkaline, neutral, and acidic conditions (Figure S23). KNO_3_‐free electrolysis resulted in negligible NH_3_ signals (Figure S24), indicating that appreciable NH_3_ formation requires the presence of nitrate. Similarly, negligible NH_3_ signals were observed under open‐circuit potential conditions (Figure S25), confirming that NH_3_ formation requires an applied potential. To directly verify the nitrogen source of the generated NH_3_, ^15^NO_3_^−^ isotope‐labeling experiments were further performed for both Cu─N_3_ and Cu─N_4_ under alkaline, neutral, and acidic conditions at ‐0.5 V vs. RHE (Figure S26). The ^1^H NMR spectra exhibit the characteristic ^15^NH_4_^+^ signals when ^15^NO_3_^−^ was used as the reactant, whereas the corresponding ^14^NH_4_^+^ signals were observed with ^14^NO_3_^−^, directly confirming that the nitrogen in the generated NH_3_ originates from nitrate. In addition, the NH_3_ concentrations independently quantified by ^1^H NMR were in good agreement with those determined using the indophenol assay, further supporting the reliability of the NH_3_ quantification.

The NH_3_ partial current density (*j*_NH3_) was further evaluated across different pH conditions to provide a complementary assessment of NH_3_ production performance (Figure 2d,e). Under alkaline conditions, Cu─N_3_ exhibits a more positive onset and maintains a greater *j*_NH3_ than Cu─N_4_ throughout the investigated potential range. The difference in *j*_NH3_ between the two catalysts becomes considerably less pronounced under neutral electrolyte and is ultimately reversed under acidic conditions, whereas Cu─N_4_ displays a more positive onset potential and higher *j*_NH3_ than Cu─N_3_. Importantly, the pH‐dependent evolution of *j*_NH3_ closely parallels that of the NH_3_ yield rate, confirming that the observed productivity trend is robust across complementary performance descriptors. To further examine the catalytic activity beyond the geometric electrode response, the electrochemically accessible surface areas (ECSA) of Cu─N_3_ and Cu─N_4_ were evaluated from the double‐layer capacitance (C_dl_) under alkaline, neutral, and acidic conditions (Figures S27–S29). The two catalysts exhibit comparable C_dl_ and ECSA values within each electrolyte. The ECSA‐normalized *j*_NH3_ (*j*_NH3, ECSA_) of Cu─N_3_ is approximately 2.78‐ and 2.58‐fold higher than that of Cu─N_4_ under alkaline and neutral conditions, respectively, whereas that of Cu─N_4_ is approximately 39.0‐fold higher than that of Cu─N_3_ under acidic conditions. These results show that ECSA normalization does not alter the distinct pH‐dependent activity relationship between Cu─N_3_ and Cu─N_4_. Figure 2f summarizes the apparent turnover frequency (TOF) of Cu─N_3_ and Cu─N_4_ across different pH conditions at ‐0.5 V vs. RHE, calculated based on the total Cu content determined by ICP‐OES. In alkaline electrolyte, Cu─N_3_ exhibits the highest TOF of 38.76 s^−1^, approximately ten times higher than that of Cu─N_4_ (∼3.37 s^−1^), supporting the higher nitrate reduction activity associated with the low‐coordinated Cu sites under alkaline conditions. Under neutral conditions, the TOF of Cu─N_3_ decreases noticeably to 1.74 s^−1^, corresponding to a reduction of nearly an order of magnitude relative to the alkaline electrolyte, while the activity difference between the two catalysts is simultaneously narrowed. In acidic electrolyte, the TOF of Cu─N_3_ further declines to around 0.01 s^−1^, whereas Cu─N_4_ retains a TOF of 0.05 s^−1^, indicating that the activity advantage associated with the low‐coordinated Cu environment toward *e‐*NO_3_RR is no longer retained under proton‐rich conditions.

To further evaluate the nitrate‐conversion behavior, the residual NO_3_^−^ concentration was quantified using the corresponding calibration curve (Figure S30), and the time‐dependent NO_3_^−^ consumption was monitored under all the investigated pH conditions (Figure S31a–c). A representative nitrogen‐balance analysis was performed at ‐0.5 V vs. RHE by comparing the consumed NO_3_^−^ with the quantified NH_3_ and NO_2_^−^. The resulting nitrogen balance was near‐closed, indicating that NH_3_ and NO_2_^−^ constitute the predominant quantified nitrogen‐containing products under the examined conditions and suggesting limited contributions from other possible nitrogen products. In addition to the nitrogen‐balance analysis, the time‐dependent NO_3_^−^ concentration profiles were further analyzed using a pseudo‐first‐order kinetic model, as commonly employed for the kinetic analysis of electrocatalytic nitrate reduction [36, 37, 38]. The NO_3_^−^ consumption can be reasonably described by the pseudo‐first‐order kinetic relationship, with the corresponding apparent rate constants (*k*_obs_) determined from the slopes of the ‐ln(C/C_0_) versus time plots (Figure S31d–f). Under alkaline conditions, Cu─N_3_ exhibits a higher *k*_obs_ than Cu─N_4_ (2.4 × 10^−3^ vs. 1.0 × 10^−3^ min^−1^), while the difference becomes less pronounced under neutral conditions (5.5 × 10^−4^ vs. 3.4 × 10^−4^ min^−1^). Under acidic conditions, Cu─N_3_ exhibits a substantially lower *k*_obs_ than Cu─N_4_ (8.3 × 10^−6^ vs. 8.5 × 10^−5^ min^−1^). These apparent nitrate‐conversion kinetics are consistent with the pH‐dependent catalytic activity observed from the NH_3_ production measurements, providing complementary kinetic evidence from the nitrate‐consumption behavior. To benchmark the catalytic performance, Cu─N_3_ and Cu─N_4_ were further compared with reported Cu─based *e‐*NO_3_RR electrocatalysts using FE_NH3_ and ICP‐normalized NH_3_ yield rate as key descriptors (Figure 2g and Figure S11). The comparison places Cu─N_3_ among representative high‐performing Cu─based catalysts under alkaline conditions, as it simultaneously achieves high FE_NH3_ and a high ICP‐normalized NH_3_ yield rate, whereas Cu─N_4_ remains comparable to previously reported Cu─based systems [14, 15, 22, 39, 40, 41, 42, 43, 44, 45, 46, 47, 48, 49, 50, 51, 52, 53, 54, 55, 56, 57, 58, 59, 60, 61, 62, 63, 64, 65, 66, 67]. To extend the electrochemical evaluation to a lower nitrate concentration, Cu─N_3_ and Cu─N_4_ were additionally tested in 0.02 m NO_3_^−^ at ‐0.5 V vs. RHE under all the investigated pH conditions (Figure S32). Compared with 0.1 m NO_3_^−^, decreasing the nitrate concentration to 0.02 m results in lower cathodic current densities and FE_NH3_ for both catalysts, likely reflecting the reduced availability of nitrate reactants at the lower concentration, while the same pH‐dependent activity relationship is observed, with Cu─N_3_ exhibiting higher FE_NH3_ than Cu─N_4_ under alkaline and neutral conditions and Cu─N_4_ exhibiting higher FE_NH3_ under acidic conditions. Given that nitrate concentrations can vary substantially among practical nitrate‐containing streams, the consistent pH‐dependent activity trend observed at 0.02 m and 0.1 m NO_3_^−^ provides an initial indication that this behavior is not restricted to high nitrate concentrations [68, 69].

Collectively, these electrochemical results reveal a pronounced pH‐dependent evolution of the coordination‐activity relationship for nitrate‐to‐ammonia conversion. The progressive attenuation and eventual inversion of the catalytic advantage of Cu─N_3_ indicate that the initial Cu coordination motif alone is insufficient to fully explain catalytic behavior across different pH environments. Instead, the observed performance trend suggests that electrolyte pH strongly regulates the transformation from the as‐prepared Cu coordination structure to the genuine working active sites. These findings therefore motivate direct investigation of the structural and electronic evolution of Cu sites under operating conditions to elucidate the origin of the observed pH‐dependent catalytic behavior.

### 2.3 In Situ XAS Deciphers pH‐Dependent Dynamic Evolution of Cu Single‐Atom Sites

In situ XAS provides a powerful approach to probe catalytic‐site evolution under electrochemical operating conditions [70, 71]. To elucidate the real structural evolution of Cu single‐atom sites during nitrate electroreduction, in situ Cu K‐edge XAS was performed under acidic, neutral, and alkaline NO_3_RR conditions. The XANES spectra collected at open‐circuit potential (OCP) and ‐0.7 V vs. RHE show distinct pH‐dependent responses for Cu─N_4_ and Cu─N_3_ (Figure 3a,b). For Cu─N_4_, pronounced spectral changes are observed under all electrolytes (Figure 3a), indicating that the initially symmetric Cu─N_4_ coordination is highly labile under *e‐*NO_3_RR conditions. Notably, the spectral evolution becomes faster and more intense as the pH decreases, suggesting accelerated Cu─site reduction and coordination reconstruction in lower‐pH environments. Consistent with this observation, linear fitting reveals a significant decrease in the average Cu valence during *e‐*NO_3_RR, with the most substantial reduction occurring under acidic conditions (Figure S12a–c). In contrast, Cu─N_3_ exhibits obvious XANES evolution mainly under alkaline conditions, whereas only limited changes are observed under neutral and acidic conditions, indicating a more regulated and pH‐selective reduction pathway (Figure 3b). The corresponding valence analysis similarly indicates a noticeable decrease in the Cu oxidation state only in alkaline media, while the average valence remains largely unchanged under neutral and acidic conditions (Figure S12d–f). These results suggest that the dynamic evolution of Cu single‐atom sites is strongly influenced by both the local coordination environment and the electrolyte pH, leading to fundamentally different reconstruction behaviors between Cu─N_4_ and Cu─N_3_ during NO_3_RR.

**FIGURE 3:**
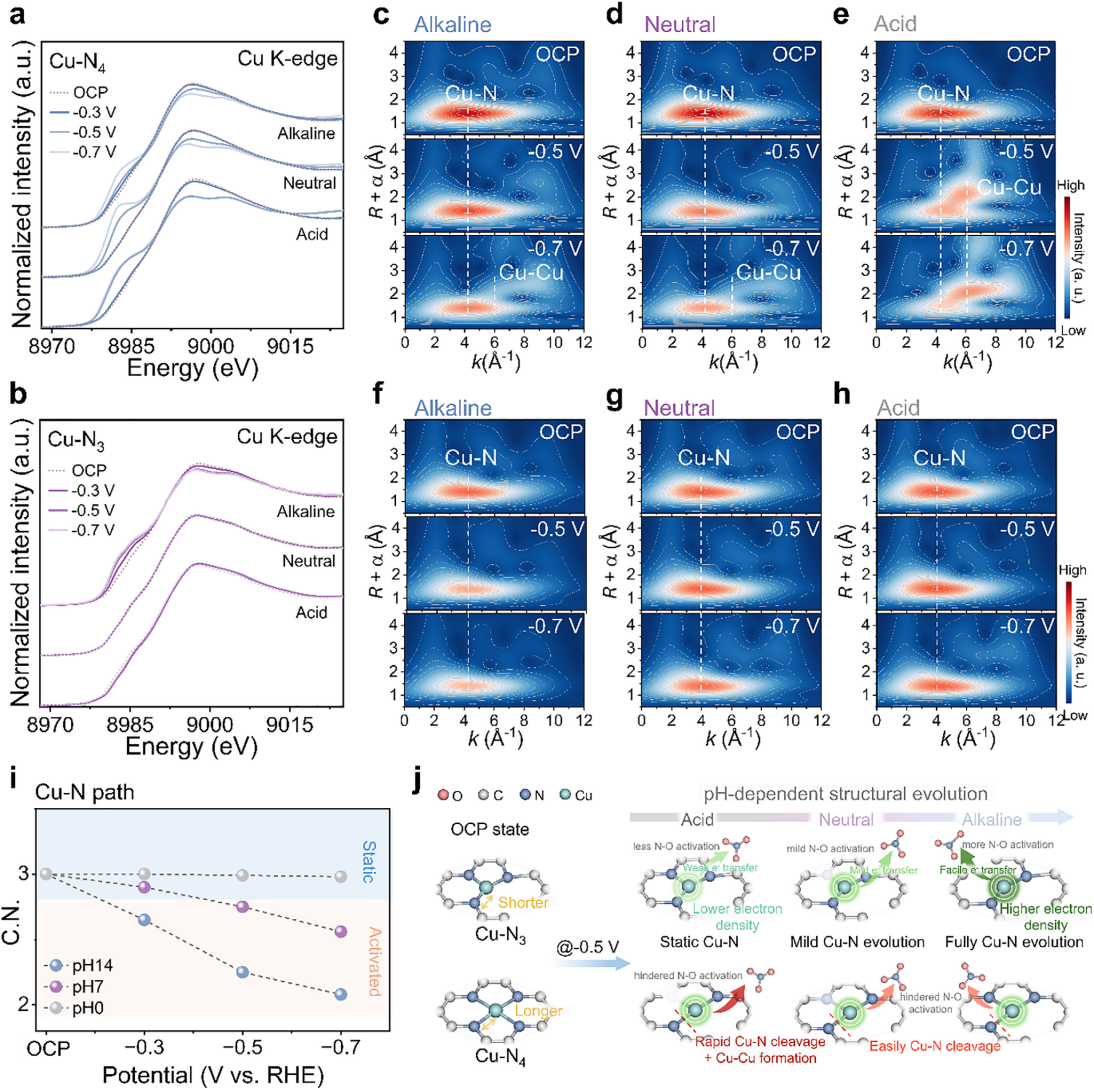
In situ evolution of Cu single‐atom sites during nitrate reduction. (a, b) in situ Cu K‐edge XANES spectra of Cu‐N_4_ and Cu‐N_3_ under various applied potentials across different pH conditions. (c‐h) in situ WT‐EXAFS contour plots of Cu‐N_4_ (c‐e) and Cu‐N_3_ (f‐h) across different pH conditions. (i) Evolution of the Cu‐N coordination environment in Cu‐N_3_ across different pH conditions. (j) Schematic illustration of the pH‐dependent dynamic structural evolution of Cu single‐atom sites during nitrate reduction.

The local coordination evolution was further resolved by WT‐EXAFS (Figure 3c–h and Figure S13). Cu─N_4_ initially exhibits an identical Cu─N scattering feature with a k‐center at approximately 4.4 Å^−1^ under alkaline, neutral, and acidic electrolytes, suggesting a nearly identical initial Cu coordination environment across the investigated electrolytes. Upon applying ‐0.5 V vs. RHE, the Cu─N scattering feature gradually attenuates under all investigated pH conditions, suggesting progressive Cu─site coordination reconstruction (Figure 3c–e). Such potential‐induced of Cu─N coordination are consistent with recent evidence that proton‐derived adsorbed hydrogen (H) can weaken Cu─N bonding under cathodic conditions, providing a mechanistic basis for the sensitivity of Cu─N coordination to the proton environment [72]. With further potential progression to ‐0.7 V vs. RHE, a distinct Cu─Cu scattering feature emerges under all investigated electrolyte conditions, becoming most pronounced in acidic electrolyte. This observation confirms that the initially isolated Cu─N_4_ sites undergo substantial reconstruction and form Cu─Cu‐containing species during NO_3_RR. This result indicates that the weakening of Cu─N coordination in the symmetric Cu─N_4_ architecture precedes pronounced Cu─site reduction and the subsequent emergence of Cu─Cu coordination, reflecting extensive structural reconstruction of Cu─N_4_. In contrast, Cu─N_3_ shows a distinctly different evolution pathway (Figure 3f–h and Figure S13d–f). A well‐defined Cu─N scattering feature centered at around 4.2 Å^−1^ is consistently observed at OCP under all investigated pH conditions. Under alkaline conditions, the Cu─N scattering feature becomes less pronounced at ‐0.5 V vs. RHE (Figure 3f), indicating progressive Cu─N coordination reconstruction and the formation of more under‐coordinated Cu─N_x_ sites. Notably, no discernible Cu─Cu scattering feature is observed throughout the investigated potential range, suggesting that Cu─N_3_ can evolve into low‐coordinated Cu species while largely preserving atomic dispersion. In neutral conditions, although changes in the Cu─N scattering feature remain subtle at ‐0.5 V vs. RHE (Figure 3g), the attenuation can still be recognized as potential progress to ‐0.7 V vs. RHE, indicating that Cu─N coordination reconstruction remains operative, but to a much lesser extent than under alkaline conditions, resulting in a more moderate evolution toward under‐coordinated Cu─N_x_ motifs. Under acidic conditions, the Cu─N scattering feature remains nearly unchanged (Figure 3h), indicating restricted coordination reconstruction and limited evolution toward under‐coordinated Cu─N_x_ motifs.

Quantitative EXAFS fitting further corroborates the pH‐dependent structural picture (Figures S14–S17 and Tables S4,S5). For Cu─N_4_, the fitted Cu─N coordination number decreases after applying potential, accompanied by the appearance of a Cu─Cu coordination shell, confirming the formation of Cu‐Cu‐containing reconstructed species (Figure S18). For Cu─N_3_, the Cu─N coordination number decreases from approximately 3 to nearly 2 under alkaline conditions, giving rise to a Cu─N_x_ configuration representative of an under‐coordinated Cu site. (Figure 3i). This coordination evolution becomes less pronounced under neutral conditions, whereas the Cu─N_3_ motif remains largely preserved under acidic conditions. To further correlate the coordination reconstruction with the electronic evolution of the Cu sites, the relative Cu─N coordination‐number change (ΔCN/CN_OCP_) was plotted against the corresponding Cu oxidation state at different applied potentials (Figure S33). The evolution toward an under‐coordinated Cu─N_x_ environment centered around a Cu─N coordination number of approximately 2 is associated with a Cu oxidation state close to +1 while maintaining atomic dispersion without discernible Cu─Cu coordination, defining a moderately reconstructed state. In contrast, further Cu─N coordination loss is accompanied by continued evolution of the Cu oxidation state toward Cu^0^ and the emergence of Cu─Cu coordination, defining excessive reconstruction associated with Cu aggregation. Notably, the more extensive evolution of Cu─N_4_ is already manifested by substantial local Cu─N coordination loss and electronic‐state changes before discernible Cu─Cu coordination emerges. Such sequential evolution is consistent with the reported dynamic behavior of isolated Cu─N sites under cathodic conditions, in which local Cu─N destabilization precedes the migration and subsequent encounter of Cu species leading to aggregation [73]. Collectively, the XANES, WT‐EXAFS, and EXAFS fitting results demonstrate that the initial Cu coordination structure governs the reduction dynamics of Cu single‐atom sites during *e‐*NO_3_RR. Whereas Cu─N_4_ undergoes rapid and excessive reconstruction toward Cu‐Cu‐containing species across the investigated pH conditions, Cu─N_3_ enables more regulated formation of under‐coordinated Cu─N_x_ sites without structural collapse, particularly under neutral and alkaline conditions.

This pH‐dependent structural evolution is consistent with the electrochemical NO_3_RR trends. Under alkaline conditions, Cu─N_3_ exhibits higher NH_3_ activity than Cu─N_4_, as reflected by its higher NH_3_ Faradaic efficiency, larger NH_3_ partial current density, and/or enhanced NH_3_ yield rate, indicating that the progressively formed under‐coordinated Cu─N_x_ sites are favorable for nitrate conversion. In contrast, although Cu─N_4_ can also generate low‐coordinated Cu species, its rapid Cu─Cu aggregation and over‐reduction limit the formation of a sustained active interface. Under neutral conditions, the activity difference between Cu─N_3_ and Cu─N_4_ becomes smaller, consistent with the moderate reconstruction of Cu─N_3_ and the partial activity contribution from Cu─N_4_‐derived reconstructed species. Under acidic conditions, Cu─N_4_ shows higher activity than Cu─N_3_, where Cu─N_4_ exhibits pronounced reconstruction involving under‐coordinated Cu─N_x_ configurations together with further evolution toward Cu‐Cu‐containing species, whereas Cu─N_3_ remains largely trapped in its less favorable initial coordination structure. To further evaluate the catalytic behavior associated with Cu─Cu‐containing structures, Cu nanoparticles were examined as a metallic Cu control under the same alkaline, neutral, and acidic conditions (Figure S34). The addition of nitrate increases the cathodic current density of the Cu nanoparticles across the investigated pH conditions, although the current difference between the nitrate‐containing and nitrate‐free electrolytes is less pronounced under acidic conditions. The FE_NH3_ follows the trend of Cu─N_3_> Cu─N_4_> Cu nanoparticles under alkaline and neutral conditions, whereas Cu─N_4_> Cu nanoparticles> Cu─N_3_ is observed under acidic conditions. Notably, the lower acidic FE_NH3_ of Cu nanoparticles compared with Cu─N_4_ indicates that the activity reversal between Cu─N_4_ and Cu─N_3_ cannot be simply attributed to the emergence of Cu─Cu‐containing coordination. Therefore, the pH‐dependent catalytic behavior should be understood as the activity of the real Cu structures generated in situ rather than a direct comparison of the initial Cu─N_3_ and Cu─N_4_ motifs. Overall, these results reveal a coordination‐modulated reconstruction window, which is summarized by the schematic illustration of Cu─site structural evolution under different pH conditions in Figure 3j. Along with this structural evolution, the increased electron density of the reconstructed Cu sites suggests more favorable interfacial electron transfer, providing an electronic contribution to the enhanced nitrate conversion. Effective *e‐*NO_3_RR requires the formation of low‐coordinated Cu─N_x_ working‐state sites to promote nitrate conversion, while avoiding Cu─Cu aggregation induced by excessive reconstruction. Insufficient reconstruction can leave Cu sites trapped in an initial coordination structure that is less favorable for *e‐*NO_3_RR, whereas excessive reconstruction disrupts the stable single‐atom active interface. Therefore, the pH‐dependent *e‐*NO_3_RR activity is essentially governed by the balance between the formation of low‐coordinated Cu─N_x_ working‐state sites and the suppression of Cu─Cu aggregation. Based on this structural picture, subsequent in situ ATR‐FTIR (Figure 4) analysis further probes how different working‐state Cu structures regulate interfacial nitrate adsorption, N─O bond activation, and the formation of nitrate‐derived intermediates.

**FIGURE 4:**
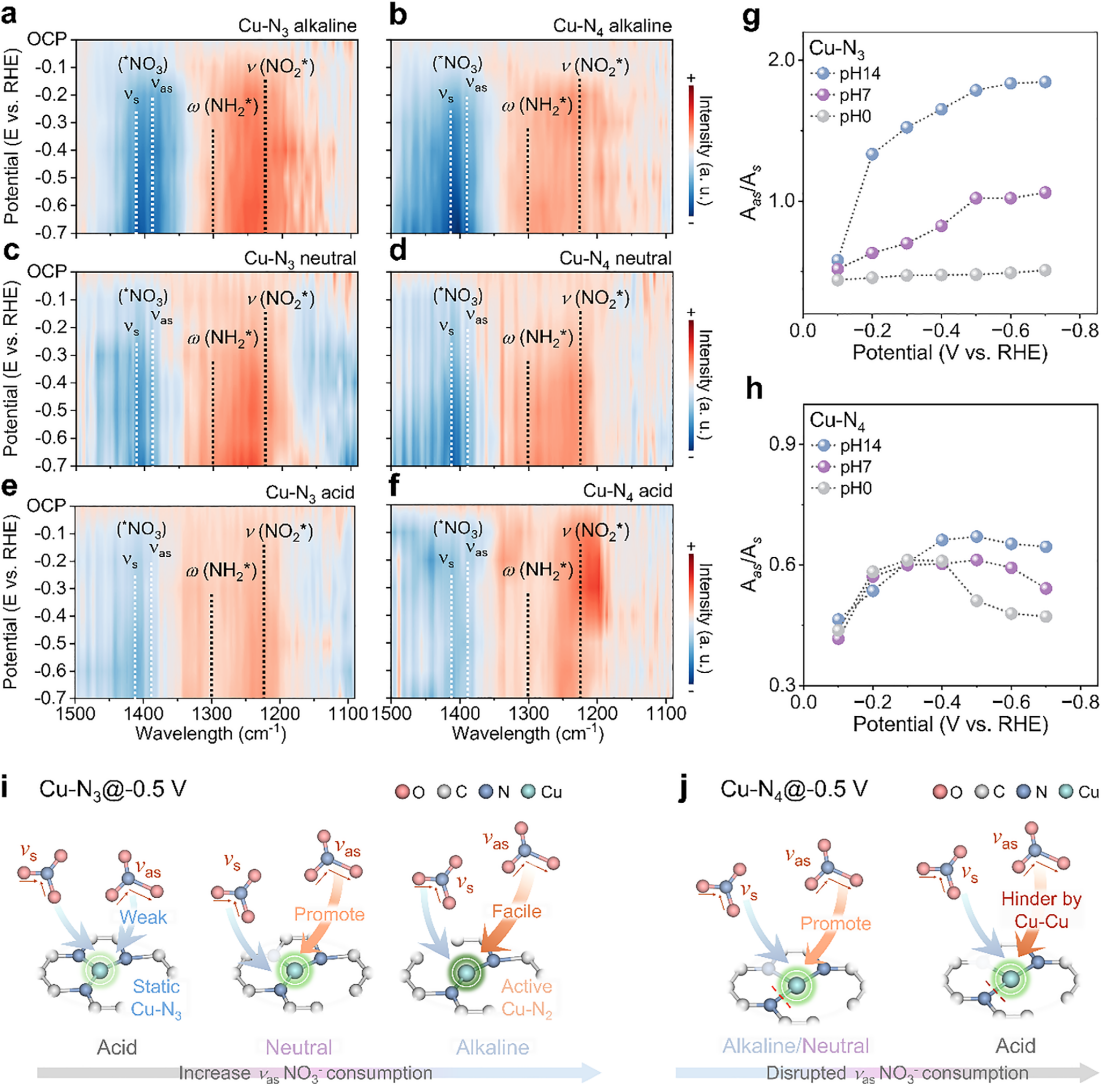
In situ ATR‐FTIR investigation of pH‐dependent interfacial nitrate reduction behavior over Cu‐N_3_ and Cu‐N_4_ catalysts. (a‐f) In situ ATR‐FTIR contour plots of Cu‐N_3_ and Cu‐N_4_ under alkaline (a, b), neutral (c, d), and acidic (e, f) conditions, respectively. (g‐h) Potential‐dependent evolution of the asymmetric‐to‐symmetric nitrate adsorption ratio (ν_as_/ν_s_) over Cu‐N_3_ and Cu‐N_4_ under different pH conditions. (i, j) Schematic illustration of pH‐dependent evolution in nitrate adsorption configurations during nitrate reduction over Cu‐N_3_ and Cu‐N_4_ catalysts.

### 2.4 In Situ FTIR Probes Interfacial Evolution in Cu SACs

To further probe how pH‐dependent coordination evolution in Cu─N_3_ and Cu─N_4_ influences interfacial nitrate adsorption and the evolution of nitrate‐derived intermediates, in situ ATR‐FTIR spectroscopy was performed over a wide pH range. Figure 4a–f presents the potential‐dependent evolution of nitrate consumption and nitrate‐derived intermediates under different electrolyte conditions. The negative bands at 1395 and 1440 cm^−1^ are assigned to the depletion of asymmetric and symmetric N─O stretching modes of adsorbed nitrate, ν_as_(N─O) and ν_s_(N─O), respectively, indicating the consumption of interfacially adsorbed nitrate species. Nitrate (NO_3_^−^) is a planar D_3_h‐symmetric anion with delocalized charge and equivalent N─O bonds. Interfacial interactions can break this symmetry, resulting in asymmetric adsorption with local N─O bond distortion and charge redistribution [74, 75]. Meanwhile, bands located at 1243 and 1300 cm^−1^ are assigned to NO_2_^*^ and NH_2_^*^, respectively, representing formation of nitrate‐derived surface intermediates based on previously reported vibrational assignments in nitrate reduction studies [70, 76]. As the pH decreases, the depletion of the nitrate bands becomes progressively less pronounced for both Cu─N_3_ and Cu─N_4_, indicating suppressed interfacial nitrate consumption and an overall attenuation of spectral evolution under proton‐rich conditions. This trend is consistent with the decreased current observed in the LSV curves. The detailed spectral evolution under different pH conditions is discussed separately in the following sections.

As shown in Figure 4a,b, Cu─N_3_ exhibits noticeable depletion of the nitrate bands beginning at approximately ‐0.2 V under alkaline conditions, whereas the corresponding nitrate‐consumption bands on Cu─N_4_ become apparent only at more negative potentials. This behavior indicates an earlier onset of interfacial nitrate consumption on Cu─N_3_, consistent with the earlier onset of NH_3_ production reflected by the *j*_NH3_ curves. With progressively more negative applied potentials, the asymmetric nitrate‐consumption band of Cu─N_3_ continues to intensify, whereas the corresponding spectral response of Cu─N_4_ increases only gradually, exhibiting limited spectral changes throughout the investigated potential range. Under neutral conditions (Figure 4c,d), Cu─N_3_ retains its advantage in initiating interfacial nitrate consumption at less negative potentials than Cu─N_4_, consistent with the earlier increase in the *j*_NH3_ curves. With progressively more negative applied potentials, the asymmetric nitrate‐consumption bands of both Cu─N_3_ and Cu─N_4_ evolve in a manner similar to that observed under alkaline conditions. Under acidic conditions (Figure 4e,f), however, this trend is reversed, with Cu─N_4_ exhibiting detectable nitrate consumption at lower overpotentials than Cu─N_3_. This behavior is accompanied by an earlier increase in the *j*_NH3_ curves. Moreover, the asymmetric nitrate‐consumption band of Cu─N_4_ becomes more pronounced than that of Cu─N_3_ throughout the investigated potential range.

To quantitatively evaluate asymmetric nitrate consumption on Cu─N_3_ and Cu─N_4_ across the pH range, the asymmetric‐to‐symmetric nitrate stretching ratio (ν_as_/ν_s_) was determined as a function of applied potential (Figure 4g,h), based on peak deconvolution of the nitrate stretching bands with the fitted values summarized in Tables S6 and S7. By normalizing the asymmetric contribution against the symmetric nitrate vibration, the *ν*_as_/*ν*_s_ ratio reduces variations in the overall nitrate‐related spectral intensity arising from differences in interfacial nitrate concentration or adsorption amount, enabling a more direct comparison of their relative vibrational contributions. As described above, the increased asymmetric contribution originates from symmetry breaking of the adsorbed nitrate, which disrupts the equivalence of the N─O bonds and induces local charge redistribution. Such an asymmetric bonding environment favors polarization of specific N─O bonds and thereby facilitates their subsequent activation [74, 75]. As shown in Figure 4g,h, under alkaline conditions, the ν_as_/ν_s_ ratio of Cu─N_3_ continuously increases from 0.58 to 1.85 with increasingly negative applied potentials, indicating progressively enhanced asymmetric nitrate consumption. In contrast, Cu─N_4_ exhibits only a gradual increase, from 0.46 to ∼0.66, suggesting a limited ability to sustain asymmetric nitrate activation at more negative potentials. Under neutral conditions, the potential‐dependent increase in the ν_as_/ν_s_ ratio is preserved but becomes considerably weaker, rising from 0.52 to 1.06 for Cu─N_3_ and from 0.42 to 0.54 for Cu─N_4_. The higher ν_as_/ν_s_ ratio observed for Cu─N_3_ under alkaline and neutral conditions reflects its pH‐dependent coordination evolution toward under‐coordinated Cu─N_x_ sites and correlates with superior nitrate conversion performance, as evidenced by the higher FE_NH3_. In contrast, although Cu─N_4_ also evolves into under‐coordinated Cu species and initially exhibits an increased FE_NH3_ with increasingly negative applied potentials, further over‐reduction induces Cu─Cu aggregation at ‐0.7 V, hindering the maintenance of a stable active interface and resulting in a decline in FE_NH3_. Under acidic conditions, however, the trend is reversed. The ν_as_/ν_s_ ratio of Cu─N_3_ remains nearly constant at approximately 0.47 throughout the investigated potential range, indicating little enhancement in asymmetric nitrate consumption. In comparison, the ν_as_/ν_s_ ratio of Cu─N_4_ increases from 0.44 to 0.61 at ‐0.3 V, followed by a gradual decrease to 0.47 at ‐0.7 V. The evolution of the ν_as_/ν_s_ ratio closely follows the trend of FE_NH3_ for Cu─N_4_. As Cu─N_4_ evolves toward a lower coordination number, both the ν_as_/ν_s_ ratio and FE_NH3_ increase, reaching their maximum values at ‐0.3 V. Beyond ‐0.5 V, the emergence of Cu─Cu coordination coincides with decreases in both the ν_as_/ν_s_ ratio and FE_NH3_, suggesting that Cu─Cu aggregation suppresses the catalytic activity. Collectively, these results demonstrate that variations in the ν_as_/ν_s_ ratio closely reflect the pH‐dependent evolution of interfacial nitrate adsorption induced by Cu─site coordination evolution and correlate well with FE_NH3_.

Overall, these results indicate that the pH‐dependent dynamic evolution of Cu coordination environments governs the persistence of asymmetric nitrate activation. Under alkaline and neutral conditions, Cu─N_3_ exhibits adaptive restructuring toward low‐coordinated Cu─N_x_ sites, enabling sustained asymmetric nitrate consumption over a broad potential window. By contrast, the transient adsorption evolution on Cu─N_4_ is terminated by rapid structural aggregation into Cu─Cu coordination at ‐0.7 V, thereby limiting the persistence of the evolved adsorption configuration. As proton availability increases under acidic conditions, the adsorption advantage of Cu─N_3_ is progressively diminished owing to the suppression of coordination evolution. Consequently, little enhancement of asymmetric nitrate adsorption is observed throughout the investigated potential range. In contrast, Cu─N_4_ undergoes coordination evolution toward under‐coordinated Cu─N_x_ sites at approximately ‐0.3 V, accompanied by increases in both the ν_as_/ν_s_ ratio and FE_NH3_. However, further reduction induces Cu─Cu aggregation, leading to declines in both asymmetric nitrate adsorption and catalytic performance. A simplified schematic of nitrate adsorption on Cu─N_3_ and Cu─N_4_ sites is illustrated in Figure 4i,j, respectively.

To further evaluate the catalytic and structural stability during prolonged operation, Cu─N_3_ and Cu─N_4_ were subjected to continuous electrolysis at ‐0.5 V vs. RHE for 10 h under alkaline, neutral, and acidic conditions (Figure S35). Cu─N_3_ maintains relatively stable NH_3_ production throughout prolonged electrolysis, with negligible decreases in both FE_NH3_ and NH_3_ yield rate under alkaline and neutral conditions, while its NH_3_ productivity remains low throughout the 10 h electrolysis under acidic conditions. In contrast, Cu─N_4_ exhibits a progressive decrease in NH_3_ production under all three conditions, with the extent of deterioration becoming more pronounced from alkaline to neutral and acidic conditions. Post‐reaction Cu K‐edge XAS further shows that Cu─N_3_ largely maintains its Cu─N coordination after 10 h of electrolysis under all three pH conditions, without the emergence of discernible Cu─Cu coordination (Figure S36 and Table S11), indicating comparatively stable local Cu coordination during prolonged operation. In contrast, Cu─Cu coordination is observed for Cu─N_4_ after 10 h of electrolysis under all three conditions (Figure S37 and Table S12), indicating that Cu─N_4_ undergoes continued reconstruction during prolonged operationICP‐OES analysis of the post‐electrolysis electrolytes further reveals nearly undetectable Cu dissolution from Cu─N_3_, whereas Cu contents of approximately 0.01 and 0.02 wt.% are detected for Cu─N_4_ under alkaline/neutral and acidic conditions, respectively, indicating a greater extent of Cu loss from Cu─N4 during prolonged operation. This continued structural evolution toward Cu─Cu‐containing structures is closely associated with the progressive deterioration in NH_3_ production, revealing distinctly different long‐term structural and catalytic stability between the two initial Cu coordination motifs. Given that sustained catalytic activity and structural integrity are critical for practical nitrate‐to‐ammonia conversion, these prolonged electrolysis and post‐reaction structural analyses further highlight the importance of regulating Cu─site reconstruction for durable *e‐*NO_3_RR operation [68, 69]. Taken together, these spectroscopic and electrochemical results establish a direct correlation between coordination evolution, electronic structure, and nitrate activation behavior. Under increasingly negative applied potentials, Cu─N_3_ sites undergo adaptive restructuring toward a catalytically favorable under‐coordinated Cu─N_x_ configuration with higher electron density, which may facilitate electron back‐donation to adsorbed nitrate. This coordination evolution enhances asymmetric nitrate adsorption, as reflected by the increased ν_as_/ν_s_ ratio, while the associated symmetry breaking and charge redistribution facilitate N─O bond activation, thereby accelerating NH_3_ production. In contrast, Cu─N_4_ undergoes further reduction leading to Cu─Cu aggregation, which results in decreased asymmetric nitrate adsorption and deteriorated catalytic performance, while the greater long‐term structural stability of Cu─N_3_ further highlights the importance of regulated Cu─site reconstruction.

## 3 Conclusion

In summary, this work demonstrates that the *e‐*NO_3_RR behavior of Cu single‐atom catalysts is governed not only by their initial coordination structures but also, more critically, by reaction‐induced coordination evolution under operating conditions. Using Cu─N_3_ and Cu─N_4_ single‐atom motifs as well‐defined pre‐catalyst models, we reveal that Cu─N_4_ undergoes rapid reconstruction across acidic, neutral, and alkaline environments. This process can generate low‐coordinated Cu species but simultaneously promotes Cu─Cu aggregation and excessive Cu─site reduction. In contrast, Cu─N_3_ exhibits more regulated site evolution, showing limited activation under acidic conditions while progressively forming Cu─N_2_‐dominated active structures under neutral and alkaline conditions. In situ XAS, WT‐EXAFS, and ATR‐FTIR analyses further establish the direct relationship among Cu coordination evolution, nitrate adsorption, N─O bond activation, and hydrogenated nitrogen intermediate formation. These findings highlight that effective Cu single‐atom catalyst design should move beyond tuning the as‐prepared electronic structure and instead consider how the initial coordination environment directs active‐site reconstruction during catalysis. This study provides a dynamic coordination strategy for promoting productive Cu─site activation while suppressing metal‐centered over‐reduction, offering mechanistic guidance for the development of pH‐adaptive Cu single‐atom catalysts for nitrate‐to‐ammonia electrocatalysis.

## 4 Experimental Section

### 4.1 Chemicals and Reagents

Copper(II) nitrate trihydrate (98%, Alfa Aesar), D‐(+)‐glucose (≥99.5%, Sigma–Aldrich), sodium chloride (99%, Seedchem), potassium nitrate (99%, Thermo Scientific), potassium hydroxide (ACS reagent, ≥85%, Sigma–Aldrich), Copper nanopowder (Alfa Aesar, 99.9%), Nafion D2020CS dispersion (20 wt.%, Chemours), salicylic acid (99%, Thermo Scientific), sulphuric acid (97%, J.T.Baker), sodium sulfate (ACS reagent, 99%, Sigma–Aldrich), trisodium citrate dihydrate (99%, Showa), sodium hydroxide (97%, Showa), sodium hypochlorite solution (10%–12%, Showa), sodium pentacyanonitrosylferrate(III) dihydrate (98%, Alfa Aesar), N‐(1‐naphthyl)ethylenediamine dihydrochloride (ACS reagent, 98%, Thermo Scientific), sulfanilamide (98%, Alfa Aesar), phosphoric acid (85 wt.% in H_2_O, 99.99% trace metals basis, Sigma–Aldrich), sulfamic acid (99.3%, ACS reagent), and hydrochloric acid (35%, Showa) were used as received without further purification. 3‐(trimethylsilyl)‐1‐propanesulfonic acid sodium salt (97%, Sigma–Aldrich), potassium nitrate‐^15^N (98 atom % ^15^N, Sigma–Aldrich)

### 4.2 Synthesis of Cu─N_4_ and Cu─N_3_ Single‐Atom Catalysts

Cu─N_4_ and Cu─N_3_ single‐atom catalysts were synthesized through a salt‐templated pyrolysis route. NaCl (4.3875 g) and D‐glucose (0.416 g) were dissolved in 25 mL of deionized water, followed by the addition of 830 mL of Cu(NO_3_)_2_ aqueous precursor solution at concentrations of 5000 and 500 ppm for Cu─N_4_ and Cu─N_3_ synthesis, respectively. The resulting precursor mixtures were frozen at ultralow temperature for 24 h and subsequently lyophilized for 48 h. The obtained dry solids were loaded into an alumina boat and subjected to an initial thermal treatment in a quartz tube furnace under flowing NH_3_ (100 mL min^−1^). The samples were heated to 300°C at a ramping rate of 5°C min^−1^ maintained for 4 h, followed by natural cooling to room temperature. The resulting solids were then dispersed in 2 L of deionized water and stirred for 24 h to leach out the NaCl template and residual byproducts. After vacuum filtration, the collected solids were dried overnight in air. A second thermal treatment was subsequently performed in a quartz tube furnace under flowing N_2_ at 850°C for 4 h with a heating rate of 5°C min^−1^ to obtain the final Cu─N_4_ and Cu─N_3_ catalysts. The Cu─free N‐doped carbon matrix were synthesis with the identical procedure while without adding a Cu precursor.

### 4.3 Characterization

The Cu content of the catalysts was quantified by inductively coupled plasma optical emission spectroscopy (ICP‐OES). Catalyst morphology and atomic dispersion were examined by scanning electron microscopy SEM (JEOL, JSM‐6700F) and aberration‐corrected transmission electron microscopy TEM (JEOL, ARM‐200FTH). High‐resolution powder X‐ray diffraction (HR‐PXRD) patterns were collected at beamline TLS BL‐01C2 of the National Synchrotron Radiation Center (NSRRC), Taiwan. Surface chemical states were analyzed by X‐ray photoelectron spectroscopy (XPS, PHI VersaProbe 4) using a monochromatic Al Ka X‐ray source under ultrahigh vacuum conditions, and all binding energies were calibrated against the C1*s* peak at 284.6 eV. Cu K‐edge X‐ray absorption spectroscopy (XAS) was collected in fluorescence mode at TPS beamline BL‐32A of NSRRC, Taiwan. The acquired XAS data analysis was conducted using the Demeter software package, with ATHENA employed for spectral processing and ARTEMIS for EXAFS structural fitting [77]. Detailed fitting procedures are provided in the Supporting Information.

### 4.4 Electrode Preparation

The catalyst ink was formulated using a solvent mixture consisting of ethanol, deionized water, and Nafion binder. To prepare the binder solution, commercial Nafion (20 wt.%) was diluted to 5 wt.% with absolute ethanol. A 20 µL aliquot of the solvent mixture, containing 1.0 mL of the diluted Nafion solution, 4.5 mL of ethanol, and 4.5 mL of deionized water, was mixed with 0.05 mg of catalyst powder and ultrasonicated until complete dispersion was achieved. The resulting suspension was deposited onto carbon cloth (1.0 cm^2^) and dried in air corresponding to a catalyst loading of 0.05 mg cm^−2^.

### 4.5 Electrochemical Measurements

Electrochemical measurements were conducted at room temperature in a two‐compartment H‐cell separated by a Nafion117 membrane. A conventional three‐electrode configuration connected to a CHI6278e electrochemical workstation (CH Instrument, USA) was employed, with platinum foil serving as the counter electrode. Hg/HgO was used as the reference electrode in alkaline electrolyte, whereas Ag/AgCl was used for measurements performed in neutral and acidic electrolytes. All the potentials reported herein were converted to the reversible hydrogen electrode (RHE) scale according to Equation (1):

(1) $$E_{\mathrm{RHE}}=E_{\mathrm{reference}}+{E^{0}}_{\mathrm{reference}}+0.059\;\mathrm{pH}$$

where E_RHE_ is the potential referenced to the reversible hydrogen electrode, E_reference_ is the measured potential versus the reference electrode, and E^0^_reference_ is the standard potential of the corresponding reference electrode (0.13 V for Hg/HgO and 0.197 V for Ag/AgCl)

Linear sweep voltammetry (LSV) was performed in 1.0 m KOH, 0.5 m Na_2_SO_4_, and 0.5 m H_2_SO_4_ electrolytes in the absence or presence of 0.1 M KNO_3_ over a potential range of 0 to ‐0.7 V vs. RHE. Unless otherwise specified, all LSV measurements were recorded at a scan rate of 5 mv s^−1^ with 90% iR compensation. Chronoamperometric measurements were carried out in electrolytes containing 0.1 M KNO_3_ under various applied potentials for 0.25 h with 90% iR compensation. Unless otherwise stated, all electrochemical measurements were performed under continuous Ar purging. The C_dl_ values were determined from the linear relationship between the capacitive current density, (j_a_ − j_c_)/2, and scan rate using cyclic voltammetry recorded within a non‐Faradaic potential window (OCP ± 50 mV) at scan rates from 20 to 100 mV s^−1^. The ECSA‐normalized NH_3_ partial current density (*j_NH3_*_ECSA_) was subsequently calculated as *j_NH3_*_ECSA_ = *j*_geo._ × C_s_/C_dl_, using a specific capacitance (C_s_) of 40 µF cm^−2^) [78].

### 4.6 Quantitative Analysis of NH_3_

Ammonium production was quantified using the indophenol blue method following electrolysis. To ensure accurate NH_3_ quantification across different electrolytes, independent calibration curves were established in 1.0 m KOH, 0.5 m Na_2_SO_4_, and 0.5 m H_2_SO_4_, respectively. For samples collected from acidic electrolyte, KOH pellets were added prior to analysis to ensure alkaline conditions for the indophenol assay. For NH_3_ analysis, the electrolyte sample was first diluted to an appropriate concentration range according to the corresponding calibration curve. Subsequently, 2 mL of the diluted sample was mixed with 2 mL of a colorimetric reagent containing 5 wt.% salicylic acid and 5 wt.% sodium citrate. Then, 1 mL of 0.05 m sodium hypochlorite solution and 0.2 mL of 1 wt.% sodium nitroprusside solution were sequentially added, followed by thorough mixing. The resulting mixture was allowed to react at room temperature for 2 h before UV–vis analysis using a HITACHI U‐3900 spectrophotometer. Absorbance spectra were recorded between 500 and 800 nm, and the absorbance at 650 nm was used to determine the NH_3_ concentration.

The NH_3_ Faradaic efficiency (FE_NH3_), NH_3_ yield rate, NH_3_ partial current density (*j*_NH3_), and NH_3_ turnover frequency (TOF_NH3_) were calculated according to Equations (2)–(5):

(2) $$FE_{\mathrm{NH}3}\left(\%\right)=\left(\left(N_{\mathrm{NH}3}\times F\times 8\right)/Q\right)\times 100\%$$

(3) $$\begin{array}{ccc} & & NH_{3}\;\mathrm{Yield}\;\mathrm{rate}\;(mg_{\mathrm{NH}3}\;m{g_{\mathrm{Cu}}}^{-1}h^{-1})\\ & & \;=\left(N_{\mathrm{NH}3}\times 17.03\times 10^{-3}\right)/(m_{\mathrm{Cu}}\times t) \end{array}$$

(4) $$j_{\mathrm{NH}3}\;\left(\mathrm{mA}\;cm^{-2}\right)=j\times FE_{\mathrm{NH}3}$$

(5) $$\mathrm{TO}F_{\mathrm{NH}3}\left(s^{-1}\right)=N_{\mathrm{NH}3}/(N_{\mathrm{Cu}\;}\times t)$$

where N_NH3_ is the amount of NH_3_ produced (mol), F is the Faraday constant (96485 C mol^−1^), Q is the total charge passed during electrolysis, m_Cu_ is the Cu loading of the catalysts, N_Cu_ is the Cu amount on the electrode (mol), *j* is obtained from the chronoamperometric current, and t is the electrolysis duration. The factor of 8 corresponds to the number of electrons transferred for the reduction of NO_3_^−^ to NH_3_, while 17.03 represents the molecular weight of NH_3_.

### 4.7 ^15^N isotope‐Labeling Experiments

To verify the nitrogen source of the produced NH_3_, ^15^N isotope‐labeling experiments were performed under alkaline, neutral, and acidic conditions using 1.0 m KOH, 0.5 m Na_2_SO_4_, and 0.5 m H_2_SO_4_, respectively, each containing 0.1 m K^15^NO_3_. Electrolysis was conducted at ‐0.5 V vs. RHE for 0.25 h. For the alkaline and neutral samples, 1160 µL of the electrolyte collected after electrolysis was first acidified with 100 µL of 6 m H_2_SO_4_ to convert the produced NH_3_ to NH_4_^+^, whereas the acidic sample was used directly without additional acidification. Subsequently, 900 µL of each sample was mixed with 40 µL of 25 mm 3‐(trimethylsilyl)‐1‐propanesulfonic acid sodium salt (DSS) as an internal standard and 60 µL of D_2_O. A 500 µL aliquot of the resulting solution was transferred into a 5 mm NMR tube for analysis. The ^1^H NMR spectra were acquired using a JEOL ECZ500R/S1 500 MHz high‐resolution NMR spectrometer at 298 K with 2048 scans, an acquisition time of 4.78 s, and a relaxation delay of 2.5 s. The chemical shifts were referenced to DSS at 0 ppm. The NH_4_^+^ concentration was quantitatively determined from the integrated NH_4_^+^ signal relative to the DSS internal standard, taking into account the known DSS concentration and the corresponding number of contributing protons.

### 4.8 Quantitative Analysis of NO_2_^−^

Nitrite (NO_2_^−^) generated during electrolysis was quantified using the Griess colorimetric method. The Griess reagent was prepared by dissolving 80 mg of N‐(1‐naphthyl)ethylenediamine dihydrochloride and 4 mg of sulfanilamide in a solution containing 0.2 mL of H_3_PO_4_ and 1.0 mL of deionized water. After electrolysis, the catholyte was collected and appropriately diluted so that the NO_2_^−^ concentration fell within the linear range of the calibration curve. An aliquot of the diluted catholyte (2.0 mL) was then mixed with 2.0 mL of 1 M HCl to adjust the solution pH, followed by the addition of 0.08 mL of the freshly prepared Griess reagent. The resulting mixture was allowed to react at room temperature for 15 min before UV–vis spectroscopic analysis. The UV–vis spectrum was recorded over the wavelength range of 400–650 nm, and the absorbance at 540 nm was used to determine the NO_2_^−^ concentration according to the corresponding calibration curve. The Faradaic efficiency toward NO_2_^−^ (FE_NO2‐_) was calculated using Equation (4): NO_2_^−^ Faradaic Efficiency (%) = ((N_NO2‐_ ×F×2)/Q) × 100%, where N_NO2‐_ represents the amount of NO_2_^−^ produced (mol), F is the Faraday constant (96485 C mol^−^^1^), 2 is the number of electrons required for the reduction of NO_3_^−^ to NO_2_^−^, and Q is the total charge passed during electrolysis.

### 4.9 Quantitative Analysis of NO_3_^−^

The conversion of NO_3_^−^ was evaluated by monitoring the residual nitrate concentration after electrolysis at ‐0.5 V vs. RHE under different pH conditions and for various reaction durations. To obtain a measurable change in nitrate concentration, the geometric area of the working electrode was increased to 4 cm^2^ while maintaining the same catalyst loading of 0.05 mg cm^−2^. After electrolysis, the catholyte was collected and diluted 160‐fold prior to nitrate analysis. The diluted sample was first adjusted to an appropriate pH by the addition of 3 mL of 1 m HCl, followed by the addition of 0.1 mL of 0.8 wt.% sulfamic acid. The resulting solution was allowed to stand at room temperature for 15 min before UV–vis analysis. The absorption spectrum was recorded over the wavelength range of 200–300 nm, and the nitrate‐related absorbance was determined using the corrected absorbance, A = A_220_‐2A_275_, where A_220_ and A_275_ represent the absorbance values measured at 220 and 275 nm, respectively. The residual NO_3_^−^ concentration was subsequently determined according to the corresponding calibration curve. The NO_3_^−^ concentration was calculated using Equation (5): NO_3_^−^ concentration (mM) = N_NO3‐_/V, where N_NO3‐_ represents the amount of residual NO_3_^−^ and V is the volume of the catholyte.

### 4.10 In Situ XAS Measurements

In situ Cu K‐edge X‐ray absorption spectroscopy (XAS) measurements were conducted at beamline BL‐32A of the Taiwan Photon Source (TPS), National Synchrotron Radiation Research Center (NSRRC), Taiwan. A custom‐built Teflon electrochemical cell was employed for the in situ measurements. The incident X‐ray beam passed through the Kapton window and electrolyte before the fluorescence signal was collected by the detector. The electrochemical configuration consisted of a catalyst‐coated carbon cloth working electrode and a platinum foil counter electrode. Hg/HgO was used as the reference electrode in alkaline electrolyte (1.0 m KOH + 0.1 m KNO_3_), whereas Ag/AgCl was employed for measurements performed in neutral (0.5 m Na_2_SO_4_ + 0.1 m KNO_3_) and acidic (0.5 m H_2_SO_4_ + 0.1 m KNO_3_) electrolytes. For working electrode fabrication, 15 mg of catalyst powder was dispersed in ethanol containing 60 µL of 5 wt.% Nafion solution and ultrasonicated for 30 min to form a uniform suspension. The resulting ink was then deposited onto carbon cloth (1 × 0.7 cm^2^) and dried under ambient conditions prior to XAS measurements.

### 4.11 In Situ ATR‐FTIR Measurements

In situ ATR‐FTIR measurements were performed using a Fourier transform infrared spectrometer (Thermo Scientific Nicolet 6700) equipped with a mercury‐cadmium‐telluride (MCT) detector and a Veemax III specular reflectance accessory (Pike Technology) operated at an incident angle of 65^°^. Prior to catalyst deposition, the reflecting surface of a 60^°^ silicon prism (2 cm diameter, Veemax) was polished with alumina paste to expose a fresh silicon surface. Catalyst electrodes were prepared by depositing an ultrasonically mixed catalyst ink containing 1 mg of catalyst and 100 µL of Nafion solution onto the polished silicon prism, followed by drying under ambient conditions. Three pieces of conductive copper tape were subsequently attached around the perimeter of the prism to provide electrical contact for electrochemical measurements. The in situ electrochemical cell was configured in a three‐electrode arrangement consisting of the catalyst‐coated silicon prism as the working electrode and a platinum foil counter electrode. Hg/HgO was employed as the reference electrode for measurements conducted in 1.0 m KOH containing 0.1 m KNO_3_, whereas Ag/AgCl was used for experiments performed in 0.5 m Na_2_SO_4_ + 0.1 m KNO_3_ and 0.5 m H_2_SO_4_ + 0.1 m KNO_3_ electrolytes. Following cell assembly, a background spectrum was collected at open‐circuit potential (OCP). Infrared spectra were subsequently acquired during a series of potentiostatic measurements from ‐0.1 to ‐0.7 V versus RHE, with each potential maintained for 2 min prior to spectral collection.

## Author Contributions

S.C.H. and Y.G.L. conceived and designed the study. S.C.H. and Y.R.L. performed the sample preparation and analyzed the overall experimental data under the supervision of Y.G.L. S.C.H. and Y.R.L. were primarily responsible for the electrochemical measurements, with support from Y.C.C. W.W.W. and Y.D.L. assisted with the TEM characterization. Y.D.L., C.K.P., Y.C.L., and C.L.C. supported the in situ XAS experiments. H.C.Y., S.C.H., and C.K.P. performed the in situ XAS data analysis. H.Y.C., C.L.C., and H.M.C. provided guidance on XAS data analysis and interpretation. All authors discussed the results and contributed to writing the manuscript. Y.G.L. was responsible for overall project management and supervision.

## Conflicts of Interest

The authors declare no conflicts of interest.

## Supporting information




**Supporting File**: advs78164‐sup‐0001‐SuppMat.docx.

## Data Availability

All data are available from the corresponding author upon reasonable request.
