# Supplementary material for: Coordination‐Asymmetry‐Regulated Reconstruction of Electron‐Rich Cu Sites for pH‐Dependent Nitrate‐to‐Ammonia Electrocatalysis
Source: Adv Sci (Weinh). 2026 Oct 14:e78164. Online ahead of print. doi: 10.1002/advs.78164 (PMC13669220; doi:10.1002/advs.78164)
Supplement: Supplementary file 1 [file ADVS-9999-e78164-s001.docx]

**Supporting Information**

Coordination-Asymmetry-Regulated Reconstruction of Electron-Rich Cu Sites for pH-Dependent Nitrate-to-Ammonia Electrocatalysis

*Shih-Ching Huang^A,†^, Yu-Ru Lin^A,†^, Hsiang-Chun Yu^B^, Yi-Dong Lin^C^,* *Yu-Chiao Chang^D^, Chun-Kuo Peng^A^, Yu-Chang Lin^E^,* *Wen-Wei Wu^C^,* *Hao Ming Chen^B^,* *Chi-Liang Chen^E^,* *Han-Yi Chen^D^, Yan-Gu Lin^A,C*^*

A. Shih-Ching Huang, Yu-Ru Lin, Chun-Kuo Peng, Yu-Chang Lin*,* Yan-Gu Lin

Scientific Research Division, National Synchrotron Radiation Research Center, Hsinchu 300092, Taiwan.

B. Hsiang-Chun Yu, Hao Ming Chen

Department of Chemistry, National Taiwan University, Taipei, Taiwan

C. Yi-Dong Lin, Wen-Wei Wu, Yan-Gu Lin

Department of Materials Science and Engineering, National Yang Ming Chiao Tung University, Hsinchu, 300093, Taiwan.

D. Yu-Chiao Chang, Han-Yi Chen

Department of Materials Science and Engineering, National Tsing Hua University, Hsinchu, Taiwan

E. Yu-Chang Lin, Chi-Liang Chen

Experimental Facility Division, National Synchrotron Radiation Research Center, Hsinchu 300092, Taiwan.

*: Corresponding: Yan-Gu Lin ([lin.yg@nsrrc.org.tw](mailto:lin.yg@nsrrc.org.tw))

†: These authors contributed equally to this work

**Figure S1.** High-resolution TEM image of the Cu-N_4_ catalyst.

**Figure S2.** High resolution powder XRD pattern of Cu-N_4_ and Cu-N_3_ catalysts.

**Table S1.** ICP-OES analysis for Cu single atom.

|  | Cu (wt. %) |
| --- | --- |
| Cu-N_4_ | 0.7670 |
| Cu-N_3_ | 0.1076 |

**Figure S3.** Corresponding Cu K-edge *k*-space EXAFS spectra for Cu-N_4_, Cu-N_3_, and relevant Cu references.

**Quantitative EXAFS Curve-Fitting Procedure**

EXAFS spectra were analyzed using the ATHENA program in the Demeter package. After post-edge background subtraction and edge-step normalization, the spectra were converted to k^2^-weighted χ(k) functions and Fourier transformed from k-space (2-10 Å^-1^) to R-space using a Hanning window (dk = 1 Å^-1^). The resulting R-space spectra enabled identification of scattering contributions from distinct coordination shells and provided structural information on the local environment of the absorbing atoms.

To obtain quantitative structural information, least-squares fitting of the EXAFS spectra was performed using the ARTEMIS module in the Demeter package. The k^2^-weighted χ(k) data were fitted in R-space over the range of 1-3 Å using scattering paths generated from the corresponding structural models. The fitting provided key structural parameters, including the coordination number (CN), interatomic distance (R), Debye-Waller factor (σ²), and edge-energy shift (ΔE_0_) for both the Cu foil reference and Cu single-atom catalysts.

The amplitude reduction factor (S_0_^2^) was first determined from fitting of the Cu foil reference (**Figure S4**), in which the coordination number was fixed at 12 while R, σ^2^, and ΔE_0_ were allowed to vary. An S_0_^2^ value of 0.9 was obtained and subsequently fixed for all Cu single-atom catalyst fittings. During the fitting of the Cu single-atom samples, both the CN and σ^2^ were constrained to positive values to ensure physically meaningful solutions and avoid intensity cancellation, whereas ΔE_0_ was treated as a free variable. Using this fitting protocol, satisfactory agreement between the experimental and fitted spectra was achieved, as shown in **Figure 1 c** and **Figure S14 to Figure S17**. The corresponding fitting parameters are summarized in **Tables S2, S4, and S5**.

**Figure S4.** EXAFS fitting result of Cu foil at Cu K-edge.

**Table S2**. Structural parameters from Cu K-edge EXAFS fits of the samples.

| Sample | Shell | CN | R (Å) | σ^2^(10^-3^Å^-2^) | ΔE_0_(eV) | *R*-factor |
| --- | --- | --- | --- | --- | --- | --- |
| Cu Metal | Cu-Cu | 12.00 | 2.54±0.01 | 8.69±0.18 | 4.77±0.23 | 0.001 |
| Cu-N_4_ | Cu-N | 4.00±0.20 | 1.93±0.01 | 8.03±0.66 | -1.15±0.37 | 0.001 |
| Cu-N_3_ | Cu-N | 3.00±0.19 | 1.91±0.01 | 5.49±0.95 | -3.07±0.63 | 0.002 |

**Table S3**. XPS fitting results of N 1*s* in Cu-N_3_, Cu-N_4_ and Cu-free N-doped carbon support (N-C) catalysts.

| Peak perentage (%) | | | | | |
| --- | --- | --- | --- | --- | --- |
|  | Prydinic N | Pyrrolic N | Graphitic N | Oxidized N | Cu-N |
| Cu-N_4_ | 16.41 | 6.31 | 68.77 | 4.04 | 4.46 |
| Cu-N_3_ | 12.88 | 6.87 | 73.82 | 4.29 | 2.15 |
| N-C | 18.08 | 6.58 | 67.76 | 7.53 | -- |

**Figure S5.** Linear fitting analysis for estimation of the Cu single-atom valence state.

**Figure S6.** Linear sweep voltammograms of Cu-N_3_, Cu-N_4_, and Cu-free carbon matrix (N-C) recorded with and without KNO_3_ under **a.** 1.0 M KOH, **b.** 0.5 M Na_2_SO_4_, and **c.** 0.5 M H_2_SO_4_ electrolyte.

**Figure S7.** Chronoamperometry curves of **a.** Cu-N_3_ and **b.** Cu-N_4_ recorded in 1.0 M KOH+0.1 M KNO_3_ under various applied potentials.

**Figure S8.** Chronoamperometry curves of **a.** Cu-N_3_ and **b.** Cu-N_4_ recorded in 0.5 M Na_2_SO_4_+0.1 M KNO_3_ under various applied potentials.

**Figure S9.** Chronoamperometry curves of **a.** Cu-N_3_ and **b.** Cu-N_4_ recorded in 0.5 M H_2_SO_4_+0.1 M KNO_3_ under various applied potentials.

**Figure S10. NH_4_^+^ quantification in different electrolyte conditions.** UV-vis absorption spectra of the indophenol assay for NH_4_^+^ quantification in **a.** 1.0 M KOH, **b.** 0.5 M Na_2_SO_4_, and **c.** 0.5 M H_2_SO_4_ over a concentration rang of 0-2.0 μg mL^-1^. Linear calibration curve for NH_4_^+^ quantification established under electrolyte condition of **d.** 1.0 M KOH, **e.** 0.5 M Na_2_SO_4_, and **f.** 0.5 M H_2_SO_4_ using the indophenol assay.

**Figure S11.** Comparison of nitrate reduction performance of Cu-N_3_ and Cu-N_4_ catalysts under acidic.

**Figure S12.** Linear fitting analysis for estimation of the *in-situ* Cu single-atom valence state of (**a-c**) Cu-N_3_ and (**d-f**) Cu-N_4_ durung *e*-NO_3_RR. **a, d.** 1.0 M KOH, **b, e.** 0.5 M Na_2_SO_4_, and **c, f.** 0.5 M H_2_SO_4_ containing 0.1 M KNO_3_.

 **Figure S13.** *In-situ* WT-EXAFS contour plots of (**a-c**) Cu-N_4_ and (**d-f**) Cu-N_3_ at -0.3 V vs. RHE under different pH conditions. **a.** 1.0 M KOH. **b.** 0.5 M Na_2_SO_4_. **c.** 0.5 M H_2_SO_4_.

**Figure S14.** Corresponding *in-situ* k-space EXAFS spectra oscillation of Cu-N_4_ under **a.**1.0 M KOH, **b.** 0.5 M Na_2_SO_4_, and **c.** 0.5 M H_2_SO_4_ during NO_3_RR.

**Figure S15.** Cu K-edge EXAFS fitting curves recorded from OCP to -0.7 V vs. RHE of Cu-N_4_ under **a.**1.0 M KOH, **b.** 0.5 M Na_2_SO_4_, and **c.** 0.5 M H_2_SO_4_ during NO_3_RR. The detailed fitting results are summarized in Supplementary Table 4.

**Figure S16.** Corresponding *in-situ* k-space EXAFS spectra oscillation of Cu-N_3_ under **a.**1.0 M KOH, **b.** 0.5 M Na_2_SO_4_, and **c.** 0.5 M H_2_SO_4_ during NO_3_RR.

**Figure S17.** Cu K-edge EXAFS fitting curves recorded from OCP to -0.7 V vs. RHE of Cu-N_3_ under **a.**1.0 M KOH, **b.** 0.5 M Na_2_SO_4_, and **c.** 0.5 M H_2_SO_4_ during NO_3_RR. The detailed fitting results are summarized in Supplementary Table 5.

**Table S4.** Summary of structural parameters obtained from fitting of the *in-situ* Cu K-edge EXAFS spectra of Cu-N_4_ during NO_3_RR.

| Sample | | Shell | CN | R (Å) | σ^2^(10^-3^Å^-2^) | ΔE_0_(eV) | *R*-factor |
| --- | --- | --- | --- | --- | --- | --- | --- |
| Alkaline | OCP | Cu-N | 4.00±0.09 | 1.93±0.01 | 8.81±0.39 | 0.13±0.17 | 0.001 |
|  | -0.3 | Cu-N | 3.53±0.14 | 1.93±0.01 | 7.76±0.64 | -0.14±0.31 | 0.002 |
|  | -0.5 | Cu-N | 3.04±0.29 | 1.93±0.01 | 6.80±0.91 | 0.22±0.43 | 0.005 |
|  | -0.7 | Cu-N | 2.49±0.11 | 1.91±0.01 | 5.50±0.64 | -0.88±0.34 | 0.006 |
|  |  | Cu-Cu | 1.11±0.11 | 2.54±0.01 |  |  |  |
| Neutral | OCP | Cu-N | 4.00±0.10 | 1.93±0.01 | 8.93±0.40 | 0.83±0.19 | 0.001 |
|  | -0.3 | Cu-N | 3.50±0.12 | 1.93±0.01 | 7.28±0.55 | -0.53±0.29 | 0.001 |
|  | -0.5 | Cu-N | 2.89±0.27 | 1.91±0.01 | 7.64±1.56 | -1.23±0.78 | 0.009 |
|  | -0.7 | Cu-N | 2.03±0.11 | 1.90±0.01 | 4.45±0.71 | 0.80±0.41 | 0.009 |
|  |  | Cu-Cu | 0.89±0.10 | 2.53±0.01 |  |  |  |
| Acid | OCP | Cu-N | 4.00±0.27 | 1.93±0.01 | 9.35±1.24 | -0.36±0.57 | 0.005 |
|  | -0.3 | Cu-N | 3.49±0.11 | 1.93±0.01 | 7.50±0.54 | 0.19±0.82 | 0.002 |
|  | -0.5 | Cu-N | 2.55±0.26 | 1.90±0.01 | 8.03±0.95 | -1.79±0.67 | 0.018 |
|  |  | Cu-Cu | 2.60±0.26 | 2.54±0.01 |  |  |  |
|  | -0.7 | Cu-N | 2.12±0.24 | 1.90±0.01 | 7.73±0.77 | -4.56±0.71 | 0.012 |
|  |  | Cu-Cu | 2.82±0.22 | 2.53±0.01 |  |  |  |

**Table S5.** Summary of structural parameters obtained from fitting of the *in-situ* Cu K-edge EXAFS spectra of Cu-N_3_ during NO_3_RR.

|  | | Shell | CN | R (Å) | σ^2^(10^-3^Å^-2^) | ΔE_0_(eV) | *R*-factor |
| --- | --- | --- | --- | --- | --- | --- | --- |
| Alkaline | OCP | Cu-N | 3.00±0.12 | 1.91±0.01 | 6.28±1.00 | -1.86±0.54 | 0.003 |
|  | -0.3 | Cu-N | 2.65±0.20 | 1.91±0.01 | 6.07±1.22 | -1.58±0.65 | 0.004 |
|  | -0.5 | Cu-N | 2.25±0.18 | 1.91±0.01 | 4.75±1.26 | -1.13±0.68 | 0.004 |
|  | -0.7 | Cu-N | 2.08±0.18 | 1.91±0.01 | 3.87±1.26 | -1.03±0.72 | 0.004 |
| Neutral | OCP | Cu-N | 3.00±0.23 | 1.91±0.01 | 5.98±1.12 | -1.09±0.56 | 0.002 |
|  | -0.3 | Cu-N | 2.90±0.30 | 1.91±0.01 | 5.53±1.52 | -1.22±0.79 | 0.005 |
|  | -0.5 | Cu-N | 2.75±0.28 | 1.91±0.01 | 5.13±1.57 | -0.42±0.79 | 0.006 |
|  | -0.7 | Cu-N | 2.56±0.28 | 1.91±0.01 | 3.99±1.72 | -0.98±0.94 | 0.007 |
| Acid | OCP | Cu-N | 3.00±0.09 | 1.91±0.01 | 6.69±1.11 | -0.18±0.51 | 0.003 |
|  | -0.3 | Cu-N | 3.00±0.13 | 1.91±0.01 | 6.38±0.96 | -0.76±0.49 | 0.002 |
|  | -0.5 | Cu-N | 2.99±0.15 | 1.91±0.01 | 6.27±1.27 | -0.17±0.61 | 0.004 |
|  | -0.7 | Cu-N | 2.98±0.32 | 1.91±0.01 | 5.43±1.61 | -2.04±0.96 | 0.008 |

**Figure S18.** Evolution of the Cu-N and Cu-Cu coordination environment in Cu-N_4_ across different pH conditions.

**Table S6.** Symmetric and asymmetric peak of NO_3_^-^ stretching band areas, extracted from *in-situ* ATR-FTIR spectra of Cu-N_3_ in the range 1300-1450 cm^-1^.

| Sample | pH14 | | pH7 | | pH0 | |
| --- | --- | --- | --- | --- | --- | --- |
|  | Area_asym_ | Area_sym_ | Area_asym_ | Area_sym_ | Area_asym_ | Area_sym_ |
| -0.1 V | 0.018 | 0.031 | 0.015 | 0.029 | 0.011 | 0.025 |
| -0.2 V | 0.056 | 0.042 | 0.024 | 0.038 | 0.016 | 0.034 |
| -0.3 V | 0.064 | 0.042 | 0.035 | 0.050 | 0.017 | 0.036 |
| -0.4 V | 0.076 | 0.046 | 0.042 | 0.051 | 0.019 | 0.040 |
| -0.5 V | 0.084 | 0.047 | 0.050 | 0.049 | 0.022 | 0.046 |
| -0.6 V | 0.090 | 0.049 | 0.050 | 0.049 | 0.026 | 0.055 |
| -0.7 V | 0.096 | 0.052 | 0.052 | 0.049 | 0.027 | 0.056 |

**Table S7.** Symmetric and asymmetric peak of NO_3_^-^ stretching band areas, extracted from *in-situ* ATR-FTIR spectra of Cu-N_4_ in the range 1300-1450 cm^-1^.

| Sample | pH14 | | pH7 | | pH0 | |
| --- | --- | --- | --- | --- | --- | --- |
|  | Area_asym_ | Area_sym_ | Area_asym_ | Area_sym_ | Area_asym_ | Area_sym_ |
| -0.1 V | 0.013 | 0.028 | 0.015 | 0.036 | 0.014 | 0.030 |
| -0.2 V | 0.030 | 0.056 | 0.018 | 0.042 | 0.028 | 0.048 |
| -0.3 V | 0.040 | 0.066 | 0.027 | 0.056 | 0.030 | 0.049 |
| -0.4 V | 0.049 | 0.074 | 0.044 | 0.073 | 0.024 | 0.040 |
| -0.5 V | 0.057 | 0.085 | 0.049 | 0.080 | 0.024 | 0.046 |
| -0.6 V | 0.060 | 0.092 | 0.051 | 0.086 | 0.023 | 0.048 |
| -0.7 V | 0.071 | 0.110 | 0.052 | 0.096 | 0.025 | 0.053 |

**Table S8**. Elemental compositions of Cu-N_3_, Cu-N_4_ and Cu-free N-doped carbon support (N-C) determined by XPS.

|  | Composition (at%) | | |
| --- | --- | --- | --- |
|  | C | N | Cu |
| Cu-N_4_ | 94.7 | 4.9 | 0.4 |
| Cu-N_3_ | 96.5 | 3.4 | 0.1 |
| N-C | 96.6 | 3.4 | N.A. |

**Figure S19.** C 1*s* XPS spectra of Cu-N_3_, and Cu-N_4_ and Cu-free N-doped carbon support (N-C).

**Figure S20.** Deconvoluted N 1*s* XPS spectra of Cu-free N-doped carbon support (N-C).

**Table S9**. total charge passage and the resulting NH_3_ amount obtained under each potential of Cu-N_3_ under various pH conditions.

|  | Potential (V vs. RHE) | | | | | |
| --- | --- | --- | --- | --- | --- | --- |
|  | -0.2 | -0.3 | -0.4 | -0.5 | -0.6 | -0.7 |
| Alkaline | | | | | | |
| Q (C) | 0.469 | 2.286 | 9.873 | 27.509 | 39.147 | 88.612 |
| NH_3_ (μmol) | 0.158 | 1.519 | 9.642 | 29.521 | 43.016 | 86.122 |
| Neutral | | | | | | |
| Q (C) | 0.312 | 0.723 | 0.911 | 3.138 | 5.165 | 8.375 |
| NH_3_ (μmol) | 0.119 | 0.349 | 0.762 | 2.891 | 4.961 | 8.181 |
| Acid | | | | | | |
| Q (C) | 0.030 | 0.039 | 0.055 | 0.235 | 1.049 | 4.557 |
| NH_3_ (μmol) | 0.025 | 0.024 | 0.024 | 0.029 | 0.048 | 0.166 |

**Table S10**. total charge passage and the resulting NH_3_ amount obtained under each potential of Cu-N_4_ under various pH conditions.

|  | Potential (V vs. RHE) | | | | | |
| --- | --- | --- | --- | --- | --- | --- |
|  | -0.2 | -0.3 | -0.4 | -0.5 | -0.6 | -0.7 |
| Alkaline | | | | | | |
| Q (C) | 0.431 | 1.143 | 5.139 | 17.092 | 29.946 | 62.698 |
| NH_3_ (μmol) | 0.035 | 0.494 | 4.462 | 18.291 | 31.121 | 61.191 |
| Neutral | | | | | | |
| Q (C) | 0.593 | 0.800 | 0.947 | 1.629 | 2.147 | 2.752 |
| NH_3_ (μmol) | 0.058 | 0.047 | 0.232 | 1.080 | 1.348 | 1.754 |
| Acid | | | | | | |
| Q (C) | 0.173 | 0.335 | 0.443 | 0.674 | 2.340 | 8.228 |
| NH_3_ (μmol) | 0.083 | 0.190 | 0.239 | 0.277 | 0.391 | 0.672 |

**Figure S21. NO_2_^-^ quantification under different electrolyte conditions.** UV-vis absorption spectra of the Griess assay for NO_2_^-^ quantification in **a.** 1.0 M KOH over a concentration range of 0-0.3 mg mL^-1^, and in **b.** 0.5 M Na_2_SO_4_ and **c.** 0.5 M H_2_SO_4_ over a concentration range of 0-0.2 mg mL^-1^. Corresponding linear calibration curves for NO_2_^-^ quantification established in **d.** 1.0 M KOH, **e.** 0.5 M Na_2_SO_4_, and **f.** 0.5 M H_2_SO_4_ using the Griess assay.

**Figure S22. NO_2_^-^ Faradaic efficiency of Cu SAC catalysts under a wide pH range.** NO_2_^-^ Faradaic efficiency (FE_NO2-_) of Cu-N_3_ and Cu-N_4_ quantified from -0.2 V to -0.7 V vs. RHE in **a.** 1.0 M KOH, **b.** 0.5 M Na_2_SO_4_, and **c.** 0.5 M H_2_SO_4_ electrolytes containing 0.1 M KNO_3_.

**Figure S23.** UV-vis spectra for NH_4_^+^ detection using the indophenol blue method before electrolysis and after electrolysis with Cu-N_3_ and Cu-N_4_ under **a.** alkaline, **b.** neutral, and **c.** acidic conditions..

**Figure S24.** UV-vis spectra for NH_4_^+^ detection using the indophenol blue method after electrolysis with Cu-N_3_ at -0.7 V vs. RHE in KNO_3_-containing and NO_3_^-^-free electrolytes under **a.** alkaline, **b.** neutral, and **c.** acidic conditions.

**Figure S25.** UV-vis spectra for NH_4_^+^ detection using the indophenol blue method after the OCP control experiment with Cu-N_3_ under alkaline, neutral, and acidic conditions.

**Figure S26.** ^1^H NMR spectra of ^14^NH_4_^+^ and ^15^NH_4_^+^ generated from ^14^NO_3_^-^ and ^15^NO_3_^-^, respectively, over **a.** Cu-N_3_ and **b.** Cu-N_4_ under alkaline conditions. **c.** Comparison of NH_3_ concentrations quantified by ^1^H NMR and the indophenol blue method. **d-f.** Corresponding ^1^H NMR spectra and quantitative comparison under neutral conditions, and **g-i.** under acidic conditions.

**Figure S27.** Cyclic voltammetry (CV) curves of **a.** Cu-N_3_ and **b.** Cu-N_4_ recorded at different scan rates in the non-Faradaic region under alkaline conditions. **c.** Estimated electrochemically surface area (ECSA) derived from the double-layer capacitance (C_dl_). **d.** ECSA-normalized NH_3_ partial current density (*j*_NH3,ECSA_) at -0.5 V vs. RHE.

**Figure S28.** CV curves of **a.** Cu-N_3_ and **b.** Cu-N_4_ recorded at different scan rates in the non-Faradaic region under neutral conditions. **c.** ECSA derived from the double-layer capacitance (C_dl_). **d.** ECSA-normalized NH_3_ partial current density (*j*_NH3,ECSA_) at -0.5 V vs. RHE.

**Figure S29.** CV curves of **a.** Cu-N_3_ and **b.** Cu-N_4_ recorded at different scan rates in the non-Faradaic region under acidic conditions. **c.** ECSA derived from the double-layer capacitance (C_dl_). **d.** ECSA-normalized NH_3_ partial current density (*j*_NH3,ECSA_) at -0.5 V vs. RHE.

**Figure S30.** **NO_3_^-^ quantification under different electrolyte conditions.** UV-vis absorption spectra for NO_3_^-^ quantification in **a.** 1.0 M KOH, **b.** 0.5 M Na_2_SO_4_, and **c.** 0.5 M H_2_SO_4_ over a concentration range of 0-0.3 mg mL^-1^. Corresponding linear calibration curves for NO_3_^-^ quantification established under **d.** 1.0 M KOH, **e.** 0.5 M Na_2_SO_4_, and **f.** 0.5 M H_2_SO_4_.

**Figure S31.** Time-dependent concentration profiles of NO_3_^-,^ NO_2_^-^, and NH_3_ for Cu-N_3_ and Cu-N_4_ under **a.** alkaline, **b.** neutral, and **c.** acidic conditions. Corresponding plots of -ln(C/C_0_) as a function of reaction time under **d.** alkaline, **e.** neutral, and **f.** acidic conditions for determination of the apparent first-order rate constants (*k*_obs_).

 **Figure S32.** Linear sweep voltammograms of Cu-N_3_ and Cu-N_4_ recorded in **a.** 1.0 M KOH, **b.** 0.5 M Na_2_SO_4_, and **c.** 0.5 M H_2_SO_4_ electrolytes containing 0.02 M KNO_3_. **d.** FE_NH3_ and NH_3_ yield rate of Cu-N_3_ and Cu-N_4_ at -0.5 V vs. RHE in 1.0 M KOH, 0.5 M Na_2_SO_4_, and 0.5 M H_2_SO_4_ electrolytes containing 0.02 M KNO_3_.

**Figure S33.** Correlated evolution of the relative Cu-N coordination number (ΔCN/CN_OCP_) and Cu oxidation state for Cu-N_3_ **a-c.** and Cu-N_4_ **d-f.** at different applied potentials from the OCP state.

**Figure S34.** **a.** Linear sweep voltammograms of Cu nanoparticles recorded in the presence and absence of KNO_3_ in 1.0 M KOH, 0.5 M Na_2_SO_4_, and 0.5 M H_2_SO_4_ electrolytes. FE_NH3_ of Cu nanoparticles in **b.** 1.0 M KOH, **c.** 0.5 M Na_2_SO_4_, and **d.** 0.5 M H_2_SO_4_, each containing 0.1 M KNO_3_.

**Figure S35.** Long-term chronoamperometric stability tests of **a-c** Cu-N_3_ and **d-f** Cu-N_4_ at -0.5 V vs. RHE for 10 hours under **a, d** alkaline, **b, e** neutral, and **c, f** acidic conditions. The insets show the corresponding time-dependent NH_3_ Faradaic efficiency and yield rate.

**Figure S36.** Cu K-edge XANES spectra and EXAFS fitting curves of Cu-N_3_ recorded at OCP and after 10 h of electrolysis at -0.5 V vs. RHE under **a, b** alkaline, **c, d** neutral, and **e, f** acidic conditions. The detailed fitting results are summarized in Supplementary Table S11.

**Figure S37.** Cu K-edge XANES spectra and EXAFS fitting curves of Cu-N_4_ recorded at OCP and after 10 h of electrolysis at -0.5 V vs. RHE under **a, b** alkaline, **c, d** neutral, and **e, f** acidic conditions. The detailed fitting results are summarized in Supplementary Table S12.

**Table S11.** Summary of structural parameters obtained from fitting of the Cu K-edge EXAFS spectra of Cu-N_3_ before and after the 10 h long-term stability tests.

| Sample | | Shell | CN | R (Å) | σ^2^ (10^-3^Å^-2^) | ΔE_0_ (eV) | *R*-factor |
| --- | --- | --- | --- | --- | --- | --- | --- |
| Alkaline | OCP | Cu-N | 3.00±0.14 | 1.91±0.01 | 6.54±0.79 | -0.80±0.40 | 0.003 |
|  | After 10 hours @  -0.5 V_RHE_ | Cu-N | 2.22±0.21 | 1.91±0.01 | 5.11±1.53 | -1.71±0.92 | 0.006 |
| Neutral | OCP | Cu-N | 3.03±0.22 | 1.91±0.01 | 6.26±1.19 | -1.89±0.64 | 0.005 |
|  | After 10 hours @  -0.5 V_RHE_ | Cu-N | 2.68±0.38 | 1.90±0.01 | 4.89±2.18 | -4.50±1.50 | 0.009 |
| Acid | OCP | Cu-N | 3.02±0.10 | 1.91±0.01 | 7.11±0.53 | -0.64±0.26 | 0.001 |
|  | After 10 hours @  -0.5 V_RHE_ | Cu-N | 2.94±0.06 | 1.91±0.01 | 6.64±0.31 | -1.77±0.17 | 0.001 |

**Table S12.** Summary of structural parameters obtained from fitting of the Cu K-edge EXAFS spectra of Cu-N_4_ before and after the 10 h long-term stability tests.

| Sample | | Shell | | CN | | R (Å) | σ^2^(10^-3^Å^-2^) | ΔE_0_(eV) | *R*-factor |
| --- | --- | --- | --- | --- | --- | --- | --- | --- | --- |
| Alkaline | OCP | | Cu-N | | 4.00±0.34 | 1.93±0.01 | 9.12±1.54 | 0.42±0.65 | 0.009 |
|  | After 10 hours @-0.5 V_RHE_ | | Cu-N | | 1.87±0.08 | 1.91±0.01 | 6.04±0.38 | -0.09±0.27 | 0.002 |
|  |  |  | Cu-Cu | | 1.84±0.08 | 2.54±0.01 |  |  |  |
| Neutral | OCP | | Cu-N | | 4.00±0.09 | 1.93±0.01 | 8.86±0.40 | 0.43±0.18 | 0.001 |
|  | After 10 hours @-0.5 V_RHE_ | | Cu-N | | 2.03±0.05 | 1.90±0.01 | 7.70±0.20 | 0.03±0.15 | 0.001 |
|  |  |  | Cu-Cu | | 2.24±0.05 | 2.54±0.01 |  |  |  |
| Acid | OCP | | Cu-N | | 4.00±0.25 | 1.93±0.01 | 8.84±1.10 | 0.08±0.44 | 0.004 |
|  | After 10 hours @-0.5 V_RHE_ | | Cu-N | | 2.08±0.11 | 1.90±0.01 | 6.64±0.38 | -1.92±0.30 | 0.004 |
|  |  |  | Cu-Cu | | 2.64±0.10 | 2.53±0.01 |  |  |  |
